# *In silico* Screening of Natural Compounds as Potential Inhibitors of SARS-CoV-2 Main Protease and Spike RBD: Targets for COVID-19

**DOI:** 10.3389/fmolb.2020.599079

**Published:** 2021-01-19

**Authors:** Divya M. Teli, Mamta B. Shah, Mahesh T. Chhabria

**Affiliations:** ^1^Department of Pharmaceutical Chemistry, L. M. College of Pharmacy, Ahmedabad, India; ^2^Department of Pharmacognosy, L. M. College of Pharmacy, Ahmedabad, India

**Keywords:** ACE2, COVID-19, phytoconstituents, SARS-CoV-2, spike (S) glycoprotein, MPRO

## Abstract

Historically, plants have been sought after as bio-factories for the production of diverse chemical compounds that offer a multitude of possibilities to cure diseases. To combat the current pandemic coronavirus disease 2019 (COVID-19), plant-based natural compounds are explored for their potential to inhibit the SARS-CoV-2 (severe acute respiratory syndrome coronavirus 2), the cause of COVID-19. The present study is aimed at the investigation of antiviral action of several groups of phytoconstituents against SARS-CoV-2 using a molecular docking approach to inhibit Main Protease (Mpro) (PDB code: 6LU7) and spike (S) glycoprotein receptor binding domain (RBD) to ACE2 (PDB code: 6M0J) of SARS-CoV-2. For binding affinity evaluation, the docking scores were calculated using the Extra Precision (XP) protocol of the Glide docking module of Maestro. CovDock was also used to investigate covalent docking. The OPLS3e force field was used in simulations. The docking score was calculated by preferring the conformation of the ligand that has the lowest binding free energy (best pose). The results are indicative of better potential of solanine, acetoside, and rutin, as Mpro and spike glycoprotein RBD dual inhibitors. Acetoside and curcumin were found to inhibit Mpro covalently. Curcumin also possessed all the physicochemical and pharmacokinetic parameters in the range. Thus, phytochemicals like solanine, acetoside, rutin, and curcumin hold potential to be developed as treatment options against COVID-19.

**Graphical Abstract:**
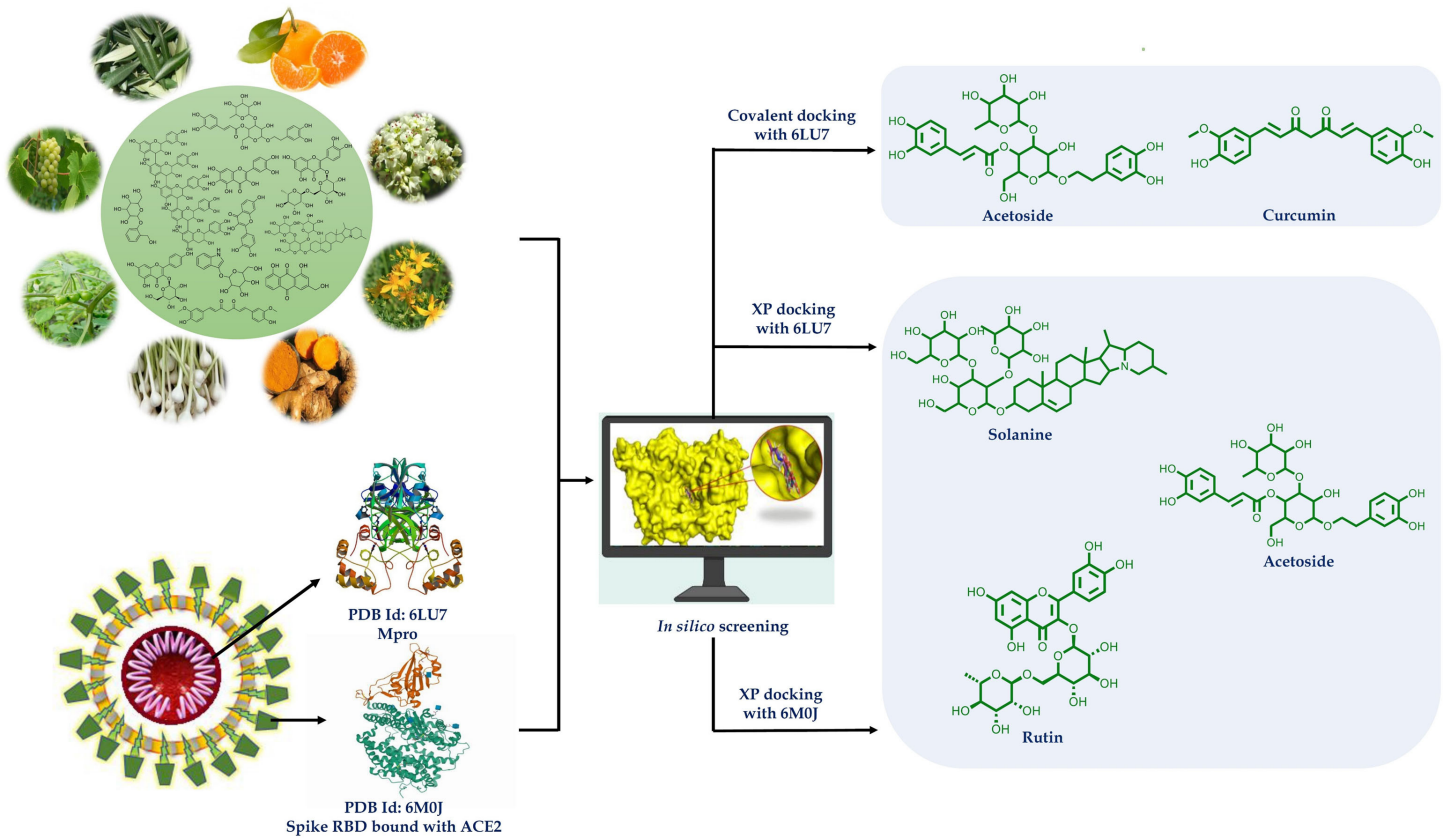


## Introduction

The World Health Organization (2020) declared coronavirus disease 2019 (COVID-19) as a pandemic, and it is affecting more than 210 countries and territories in the world. As per the WHO report (August 16, 2020), there have been 21,294,845 confirmed cases of COVID-19 and over 761,779 total deaths occurred due to it (World Health Organization, 2020). SARS-CoV-2, a member of the beta-coronaviruses (Beta-CoVs) causes a novel type of transmissible pathogenic human severe acute respiratory syndrome, characterized by symptoms of acute respiratory distress such as fever 38.1–39°C, dry cough, and shortness of breath with an incubation period of about 5 days (average 2–14 days) (Yuen et al., 2020). The human-to-human transmission of COVID-19 is reported to occur by respiratory droplets or direct contact with the patients (Jayaweera et al., 2020).

**Figure 1:**
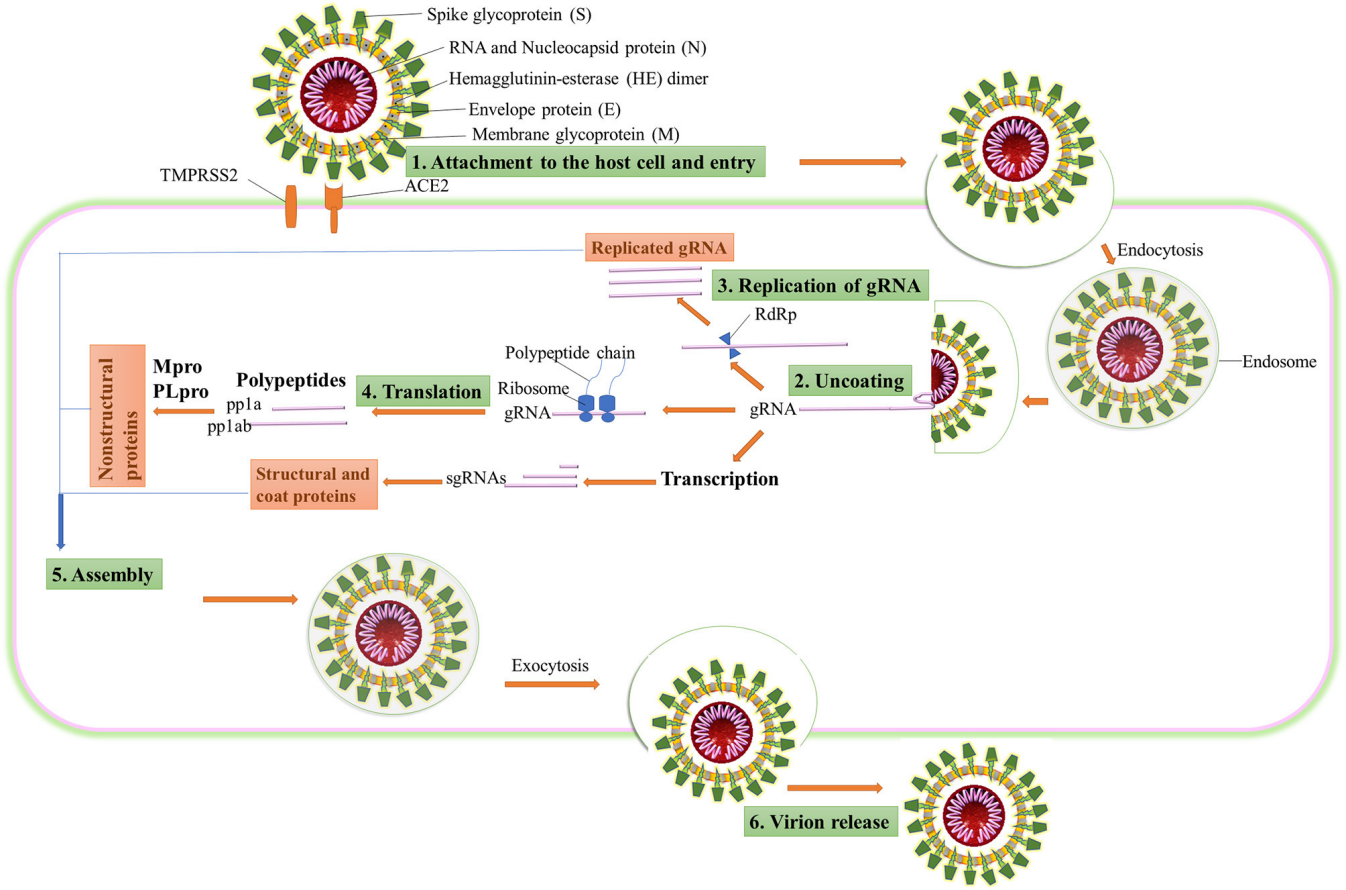
The life cycle of SARS-CoV-2 in the host cell. ACE2, angiotensin-converting enzyme 2; TMPRSS2, type 2 transmembrane serine protease; gRNA, genomic RNA; RdRP, RNA-dependent RNA polymerase; sgRNA, subgenomic RNA; pp1a, polyprotein 1a; pp1ab, polyprotein 1ab.

CoVs belong to the *Coronaviridae* family. They are a group of genotypically and phenotypically diverse, enveloped, and positive-sense viruses carrying single-stranded RNA. Although it is considered to be introduced from bats, the specific source of SARS-CoV-2, animal reservoir, and enzootic patterns of transmission remains unresolved. To understand the drug targets for COVID-19, the life cycle of SARS-CoV-2 (Figure 1) needs to be understood thoroughly. SARS-CoV-2 consists of four basic structural proteins: “spike protein (S),” “membrane (M) protein,” “envelop (E) protein,” and helically symmetrical “nucleocapsid protein (N).” The SARS-CoV-2 virus targets the host cells through the viral spike (S) protein, which binds to the ACE2 receptor of the host cells (Fung and Liu, 2014). After the S protein-ACE2 binding, the virus utilizes the host cell receptors (TMPRSS2) and enters into the cytosol of the host cell. After uncoating, the viral gRNA is released into the cytoplasm. Viral polypeptides are synthesized using the host cell protein synthesis machinery, which are further processed by viral proteases, and the products are transferred to the replicase transcriptase complex. The virus uses its RdRP to synthesize the viral RNA. Viral structural proteins and assembly proteins are also synthesized leading to the completion of the assembly and the release of progeny viral particles by exocytosis (Lu et al., 2020). β-CoVs produce pp1a and pp1ab by translation of the genomic RNA. They are proteolytically cleaved into structural and non-structural proteins by main protease (Mpro) also known as 3-chymotrypsin-like protease (3CLpro) and by papain-like protease (PLpro) (Fehr and Perlman, 2015). Once the virion assembly gets ready, it will be released from the host cell.

### Diagnostic Tools/Methods Employed in COVID-19

The detection of COVID-19 generally depends on the travel history of the person from the affected areas and analysis of their clinical symptoms. Nevertheless, asymptomatic patients may remain underdiagnosed and further contribute to the spread of the disease. In order to combat the disease progression, rapid diagnosis of SARS-CoV-2-infected patients and adherence to medical isolation are of paramount importance. Currently, three main detection strategies are available for diagnosis of COVID-19 like radiographical, amplification, and immunological methods.

Radiographical methods like Chest X-ray or computed tomography (CT) imaging was previously used in China for clinical diagnosis of COVID-19. It not only allows the diagnosis of pneumonia and acute respiratory distress syndrome but also allows early detection of pulmonary abnormalities. High-resolution computed tomography (HRCT) scans of the chest are proven as an essential tool for detection of SARS-CoV-2 at early stages. The HRCT of SARS-CoV-2-infected patients demonstrate some typical features, such as multiple peripheral bilateral hazy ground-glass opacity (GGO), pulmonary consolidation (increasing over time), bronchial inflation with diffused GGO, and thickening of the interstitium. It is found to be a great diagnostic tool for screening of COVID-19 patients especially in the high prevalence or pandemic areas. The drawbacks of CT scans include high cost, a requirement of technical experts, and inadequate specificity due to overlapping features with other viral infections or pneumonia (Borah et al., 2020). CT scans are only indicative but not confirmatory test for COVID-19.

Polymerase chain reaction (PCR) containing methods are based on the amplification of genes and their RNA transcripts isolated from biological samples. Presently, quantitative reverse transcription-polymerase chain reaction (rRT-PCR) is being used for diagnosis of COVID-19 and is a gold standard molecular diagnostic technique for many viruses as well. Single-step quantitative RT-PCR with TaqMan chemistry is more sensitive and specific. The test takes 24–48 h for the commencement of the final result.

Common laboratory findings in COVID-19 are a decreased lymphocyte count and an increased C-reactive protein (CRP) level. Serological assays are designed to detect the presence of antibodies, namely, IgM, IgG, or both using immunoassay techniques like ELISA, high-throughput chemiluminescent microparticle immunoassay (CMIA), lateral flow chromatographic immunoassay, etc. (Kaddoura et al., 2020). Based on the mentioned principles, many rapid diagnostic test kits have been developed for fast and early detection of COVID-19, which give results in 2–3 min. A throat, nasopharyngeal, and nasal swab can be used for these tests. Although it is considered that these molecular tests have 90% sensitivity, there still stands the risk and repercussions of false-negative tests with the current rapid diagnostic devices.

### Therapeutic Options for COVID-19

From earlier experience of management of such viral infections, many agents are being used as treatment options for SARS-CoV-2 viral infection. On October 22, 2020, the FDA approved remdesivir for the treatment of hospitalized patients with COVID-19. Till date (November 14, 2020), 2,932 ongoing and completed COVID-19 studies have been listed on World Health Organization's International Clinical Trials Registry Platform (https://clinicaltrials.gov/ct2/who_table). Most of the clinical trials include the use of available antiviral drugs, corticosteroids, immunomodulators, and some antimalarials alone or in combination.

Various antiviral agents have been investigated for the management of COVID-19. Apart from remdesivir, other antiviral agents like RNA-dependent RNA polymerase inhibitors (e.g., favipiravir and sofosbuvir), neuraminidase inhibitors (e.g., oseltamivir), and protease inhibitors (e.g., lopinavir and ritonavir; Mehta et al., 2020).

Corticosteroids were widely used for the treatment of SARS-CoV and MERS-CoV, and are also used in the management of the current pandemic of COVID-19 as dexamethasone, methylprednisolone, and prednisone. However, the interim guidelines by the WHO prohibit the use of routine corticosteroids unless indicated for other clinical ground. Certain interferons and immunoglobulins are also being investigated as they decrease the cytokine storm.

Chloroquine and hydroxychloroquine are found to block infection by increasing endosomal pH of the phagolysosome needed for virus/cell fusion. It also interferes with ACE2 glycosylation of SARS-CoV cellular receptors. The drug also has an immune-modulating activity, which is proposed to enhance its antiviral effect *in vivo*. Many clinical trials have been performed for checking the efficacy of chloroquine and hydroxychloroquine with or without azithromycin in COVID-19.

Convalescent plasma is an antibody-rich product made from blood donated by people who recovered from disease caused by a virus. Various clinical trials of plasma enrichment techniques have been made. The initial results were encouraging, but it is not yet approved for use by the FDA. Attempt for generation of vaccine is also in process.

Ongoing research efforts in various directions using several drug molecules against SARS-CoV-2 or related viruses have not been able to establish any type of drug to subdue the morbidity and mortality it causes (Machhi et al., 2020). There is an incessant demand for the availability and accessibility of medicines, vaccines, and diagnostics tools. The functional prominence of Mpro and spike glycoprotein in the viral life cycle, along with the lack of closely associated homologs in humans, makes them promising targets for COVID-19 antiviral drug design (Aanouz et al., 2020).

Many phytochemicals have been recorded to be used to treat infectious diseases caused by bacteria, virus, and fungi (Mahady, 2005; Ben-Shabat et al., 2020; Kumar et al., 2020). *In silico* methodologies have opened new avenues of research and have now been widely accepted as a useful tool for shortening lead times, understanding and predicting druggability in early drug discovery. Molecular docking can be used to predict how receptor protein interacts with bioactive compounds (ligands). Keeping this in view, potential bioactives from plants belonging to diverse chemical categories were assessed for the likelihood of finding suitable starting points for the lead. Thus, in the present study, flavonoids (flavones, isoflavones, flavonols, flavanone, xanthones, flavan 3-ols), tannins (hydrolysable and condensed), anthraquinones, phenolics, lignans, alkaloids, diterpenoids (limonoid, labdane), and coumarins (simple, complex) were subjected to docking studies targeting main protease (Mpro) (PDB code: 6LU7) and spike (S) glycoprotein receptor binding domain to ACE2 (PDB code: 6M0J) of SARS-CoV-2. The results of the study are indicative that procyanidin A1, A3, A4, B2, solanine, acetoside, rutin, epitheaflavin monogallate, quercitrin, and theaflavin 3,3′-digallate are Mpro and spike glycoprotein inhibitors and can be subjected to further research in finding specific regimens to overcome COVID-19. Most of these natural compounds are polyphenols chemically. Many polyphenols are reported as SARS-CoV-2 main protease inhibitors, but we have also checked here the potential of these compounds as main protease as well as spike RBD inhibitors. Some of the compounds were additionally found to inhibit main protease covalently. Further, the stability of the ligand–receptor complexes was assessed using molecular dynamic (MD) simulation studies.

## Experimental/Materials and Methodology

### Data Collection and Preparation

A total of 170 phytoconstituents belonging to the aforementioned chemical classes of secondary metabolites were shortlisted for the present study. The 3D chemical structures of the selected molecules were retrieved from PubChem. These molecules were prepared for computational study at physiological *p*H condition by using the LigPrep module of Schrödinger suite v 12.3 (Schrödinger, LLC, NY, USA, 2020). The ligand geometry was minimized by applying an OPLS3e force field algorithm (Harder et al., 2016).

### Preparation of Protein Structures

From the RCSB Protein Data Bank (https://www.rcsb.org/), the 3D structure of Mpro (PDB code: 6LU7) and spike glycoprotein RBD (PDB code: 6M0J) were obtained and prepared to ensure structural correctness for hydrogen consistency, bond orders, steric clashes, and charges using protein preparation wizard in Schrödinger suite supported by OPLS3e force field (Protein Preparation Wizard, 2020). Thus, a prepared structure was used for receptor grid generation for the docking protocol.

#### Preparation of Mpro for Docking

PDB ID 6LU7 is a 2.16-Å X-ray crystal structure of COVID-19 main protease in a complex with an inhibitor N3. The covalent bond between co-crystallized ligand N3 and protein catalytic dyad Cys145 was cleaved. After cleavage, Cys145 and ligand were reconstructed by making necessary changes and the ligand–protein complex was prepared and refined using the Protein Preparation Wizard in Schrödinger. Thus, the prepared structures were used for receptor grid generation required for the docking protocol. The receptor grid was generated on the active site of Mpro protein by considering the centroid of co-crystalized ligand molecule N3 as a center of the grid. The grid coordinates (i.e., X, Y, and Z) were −10.47, 12.23, and 68.7, respectively (Kanhed et al., 2020). This cysteine protease Mpro has 306 amino acids chain and consists of three main domains. Domain 1 is from residues 8–101, domain 2 is from 102 to 184, whereas domain 3 is from 201 to 203 amino acid sequence, connected to domain 2 by loop residues 185–200. The substrate-binding site on this viral protein is present in a cleft between domain 1 and 2 with a Cys145-His41 catalytic dyad. The major active subsites in the active site of Mpro, where the substrate binds, are defined. Thus, S1 subsite is made up of Phe140, Leu141, Asn142, His163, Glu166, and His172 amino acids. Hydrophobic S2 subsite is made up of His41, Met49, Tyr54, Met165, and Asp187. The S4 binding subsite involves Met165, Leu167, Phe185, Gln189, and Gln192 amino acids (Zhang et al., 2020).

#### Preparation of Spike Glycoprotein Receptor-Binding Domain for Docking

PDB ID 6M0J is a 2.45-Å X-ray crystal structure of SARS-CoV-2 spike receptor-binding domain (RBD) bound with ACE2. The protein was prepared using the Protein Preparation Wizard in Maestro v 12.3 (Schrödinger, LLC, NY, USA, 2020). In this protein, bond orders were assigned, water molecules were removed, and OPLS3e force field was applied to minimize the protein structure. The receptor grid was generated using receptor grid generation panel in Maestro, by selecting active site amino acid residues (Tyr449, Asn487, Gly496, Thr500, Gly502, and Tyr505) of chain A of the spike RBD. The grid coordinates (i.e., X, Y, and Z) were 204.45, 199.79, and 246.89, respectively (Lan et al., 2020). The SARS-CoV-2 RBD has a twisted five-stranded antiparallel β sheets (β1, β2, β3, β4, and β7) with short connecting helices and loops that form the core. SARS-CoV-2 RBD comprises residues Arg319–Phe541 (Kalathiya et al., 2020).

Analysis of the interaction surface of RBD-ACE2 reveals few contact points between RBD and ACE2. Tyr505 of RBD exhibits very stable hydrogen bonding suggesting an early contact point with ACE2. Additionally, Tyr449, Gln493, Gln498, Thr500, Asn501, and Gly502 show polar interactions with the ACE2 surface (Veeramachaneni et al., 2020). Thus, all these amino acids of RBD comprise a suitable binding site to target the RBD of the spike protein with suitable drug-like molecules.

### Molecular Docking and Interaction Visualization

The selected set of 170 phytoconstituents were subjected for molecular docking against Mpro (6LU7) and Spike protein RBD (6M0J) using the Extra Precision (XP) protocol of Glide docking module of Maestro (Friesner et al., 2006). The OPLS3e force field was used in simulations. Compounds were then ranked based on their docking scores that represent binding energies. Two structures (curcumin and acetoside) from the selected set were found to possess α, β-unsaturated ketone as N3 ligand. Therefore, covalent docking study was performed for these two. The ligand interactions with the active sites of the receptors were visualized using the academic version of PyMOL.

### Covalent Docking

The covalent docking protocol was applied after non-covalent molecular modeling for Mpro of SARS-CoV-2. Curcumin and acetoside contain α, β-unsaturated ketone, which acts as Michael acceptor group. PDB also contains such Michael acceptor group and showed covalent binding with Cys145. So, it was thought to check the covalent docking of these compounds with Mpro. For this, the CovDock module of Schrödinger Suite was used. In CovDock protocol, Cys145 was specified as a reactive residue in the receptor, Michael addition as reaction type, and α, β-unsaturated carbonyl group as ligand functional group represented by a SMARTS pattern [C,c]=[C,c]-[C,c,S,s]=[O] were selected (Zhu et al., 2014). The docking score was calculated by preferring the conformation of the pose that has the lowest binding free energy.

### *In silico* Physicochemical and Pharmacokinetic Parameter Prediction

The natural compounds, showing good binding affinities toward Mpro and spike RBD bound to ACE2 were investigated for their physicochemical and pharmacokinetic parameters *in silico*. The QikProp module of Maestro was used for this prediction (QikProp, 2020).

### Molecular Dynamic Simulation Study

The MD studies of best ranked compounds [curcumin (42) and solanine (4)] for Mpro and spike protein were performed for a period of 10 ns by using GROMACS 2020.1 software as per our previous report (Patel et al., 2020).

## Results and Discussion

### Molecular Docking Study

To achieve a multitarget approach and to find the potential candidate for treating COVID-19 infection, molecular docking studies were performed against two protein structures of SARS-Cov-2: (i) Main protease, Mpro (PDB ID: 6LU7) and (ii) Spike glycoprotein (PDB ID: 6M0J) in GLIDE (Grid-based Ligand Docking with Energetics) of Schrödinger suite v 12.3 (Schrödinger, 2020).

#### Mpro Docking

Bioactive phytoconstituents (total 170) docked against Mpro of SARS CoV-2 were found to exhibit very impressive docking scores. About 59 compounds have shown the glide score of −6 or less and good Mpro inhibition energy (Table 1).

**Table 1:** Chemical structures and docking results of phytoconstituents on Mpro of SARS-CoV-2.

**Comp. No**.	**Name**	**PubChem ID**	**Chemical structure**	**Docking score** **against Mpro (kcal/mol) (PDB ID: 6LU7)**	**Glide emodel (kcal/mol)**
1	Procyanidin A3	16129741	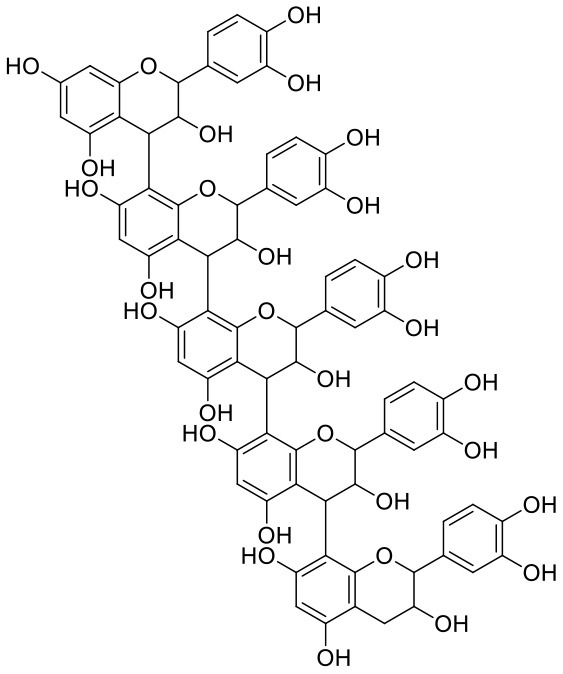	**−12.86**	**−68.210**
2	Acetoside	5281800	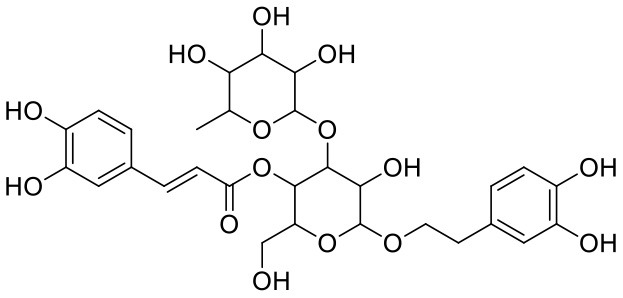	**−11.974**	**−61.801**
3	Rutin	5280805	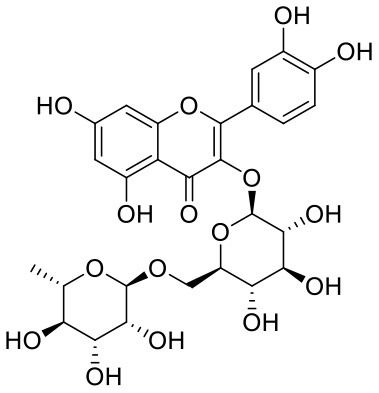	**−11.187**	**−95.135**
4	Solanine	262500	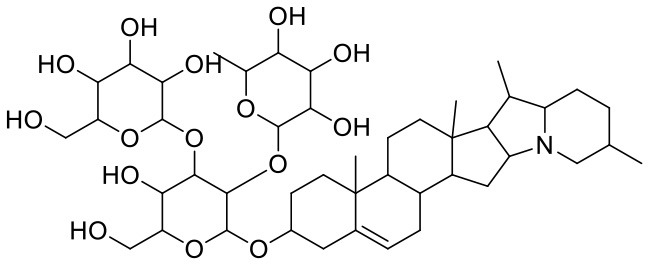	**−10.301**	**−81.460**
5	Procyanidin A4	53349182	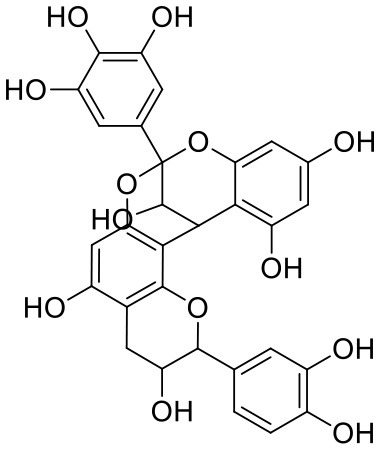	**−10.005**	**−66.051**
6	Procyanidin B4	147299	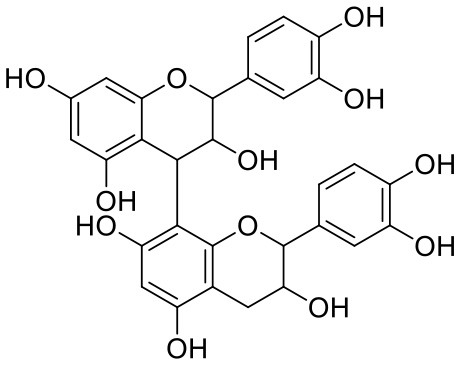	**−9.940**	**−60.339**
7	Hypericin	3663	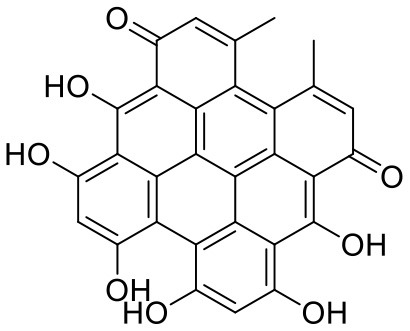	**−9.560**	**−85.277**
8	Quercetagetin	5281680	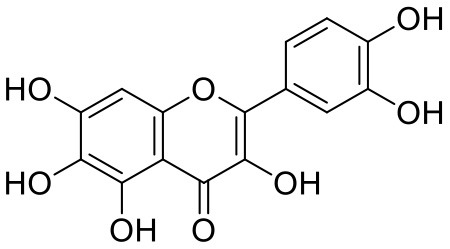	**−9.407**	**−67.987**
9	Procyanidin	107876	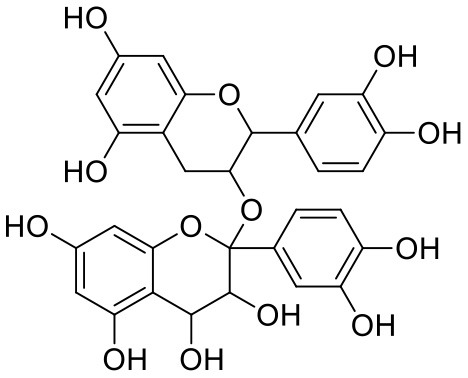	**−9.209**	**−62.543**
10	Astragalin	5282102	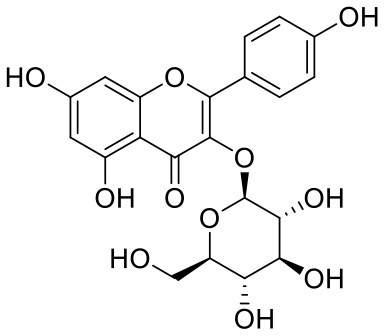	**−9.120**	**−71.431**
11	Procyanidin A1	5089889	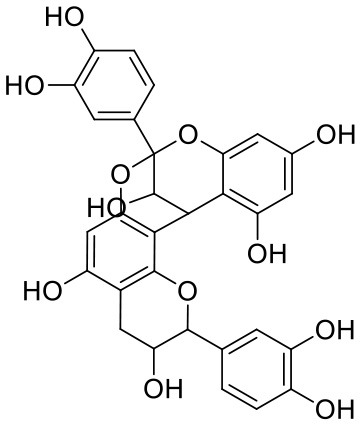	−9.204	−63.702
12	Baicalin	64982	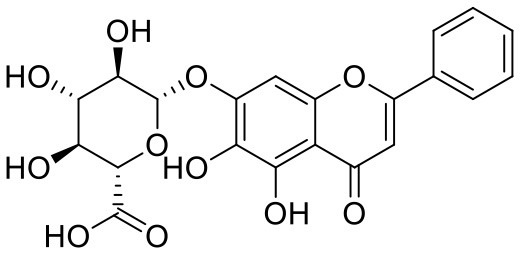	−8.818	−66.079
13	Procyanidin B2	122738	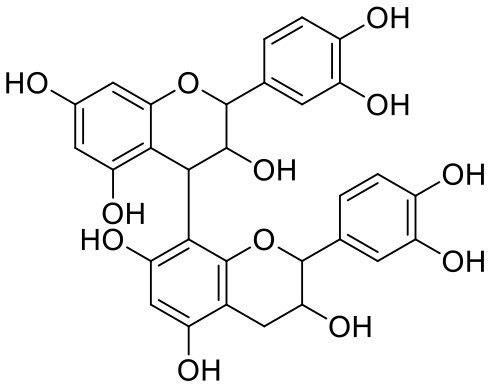	−8.557	−57.315
14	Salicin	439503	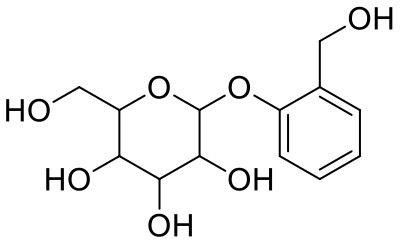	−8.448	−49.230
15	Theaflavin	135403798	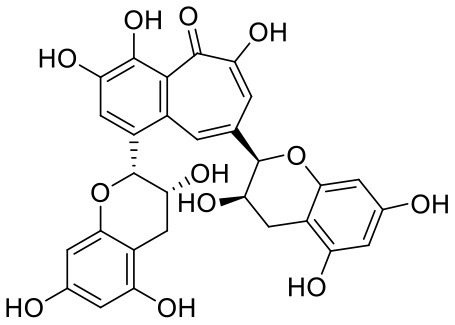	−8.383	−79.414
16	Emodin-8-glucoside	99649	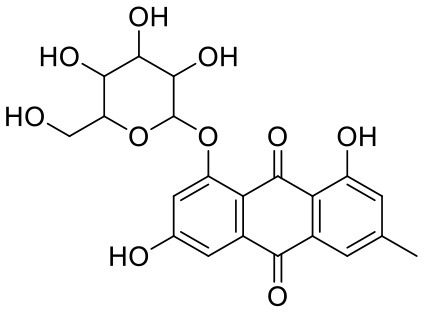	−8.211	−67.011
17	Hinokiflavone	5281627	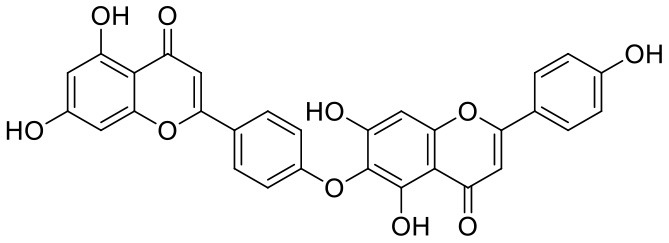	−8.130	−87.255
18	Quercitrin	5280459	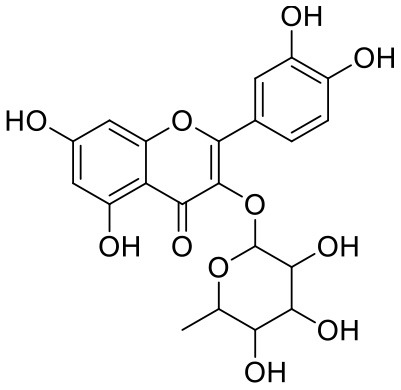	−8.121	−43.916
19	Procyanidin C2	11182062	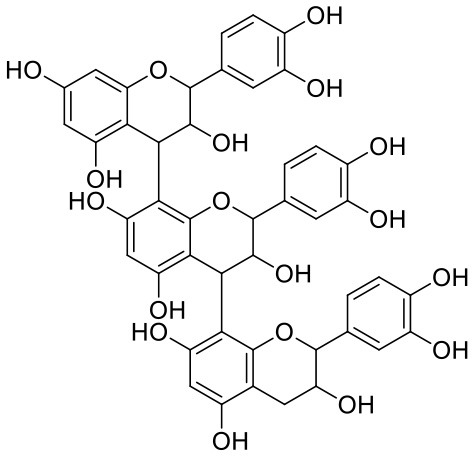	−8.107	−63.103
20	Indican	441564	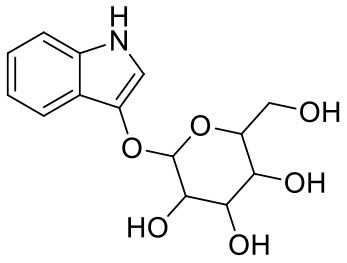	−8.084	−40.609
21	Chebulic acid	71308174	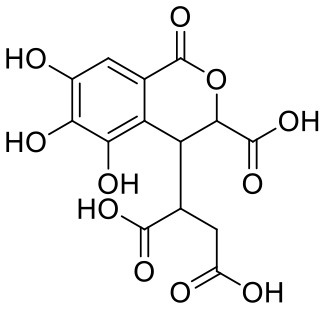	−8.077	−72.474
22	Amentoflavone	5281600	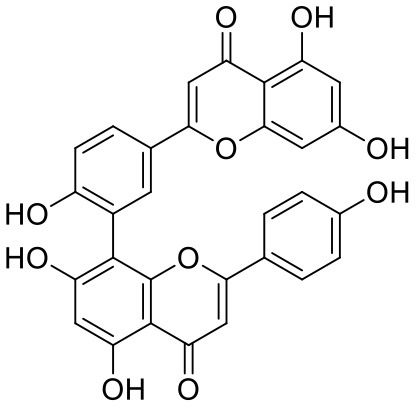	−7.981	−54.006
23	(-)-Catechin gallate	6419835	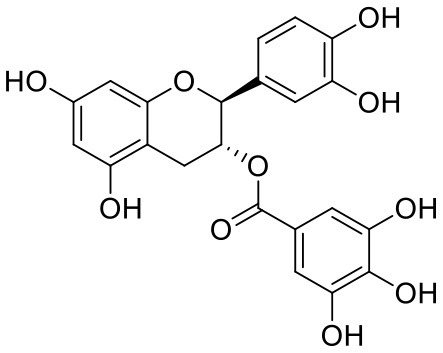	−7.956	−85.457
24	Fisetin	5281614	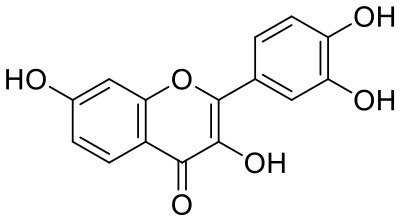	−7.940	−53.530
25	Procyanidin C1	169853	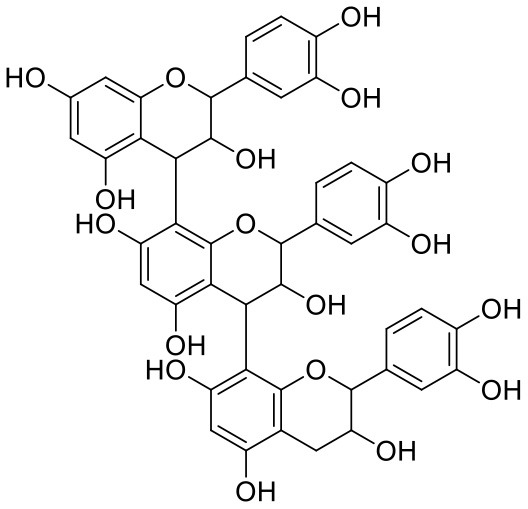	−7.814	−58.015
26	(-)-Epicatechin gallate	107905	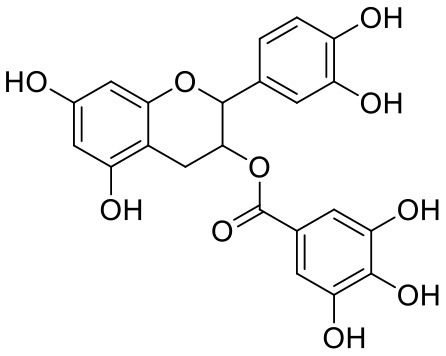	−7.857	−88.167
27	Morin	5281670	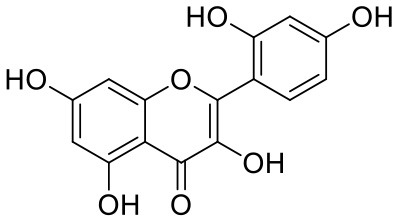	−7.631	53.217
28	Garcinol	174159	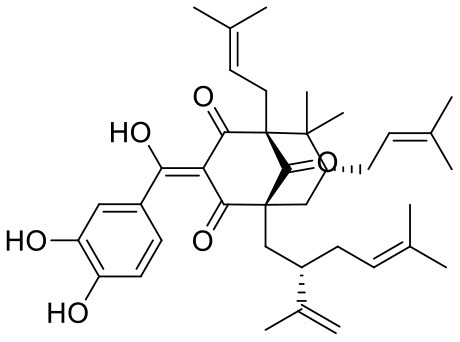	−7.551	−60.652
29	Glycyrrhizic acid	14982	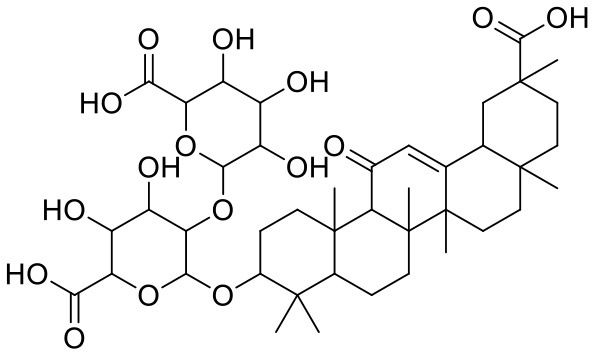	−7.549	−69.351
30	Sweroside	161036	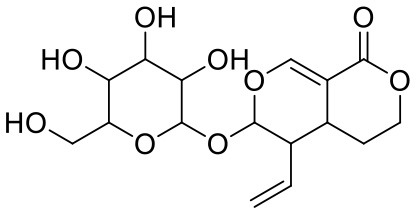	−7.547	−36.098
31	Wikstromol	99938	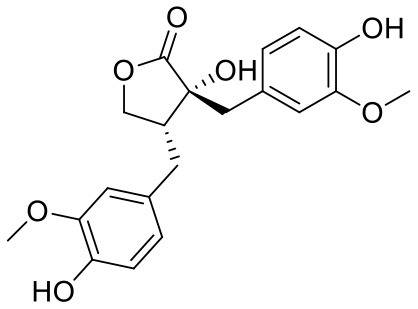	−7.547	−64.848
32	Baicalein	5281605	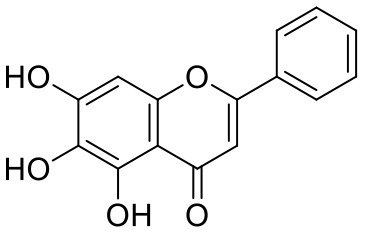	−7.463	−52.709
33	Myricetin	5281672	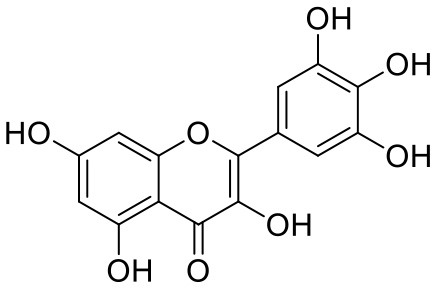	−7.311	−60.412
34	Kaempferol	5280863	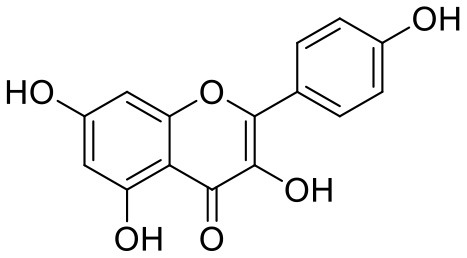	−7.307	−54.359
35	Procyanidin A2	124025	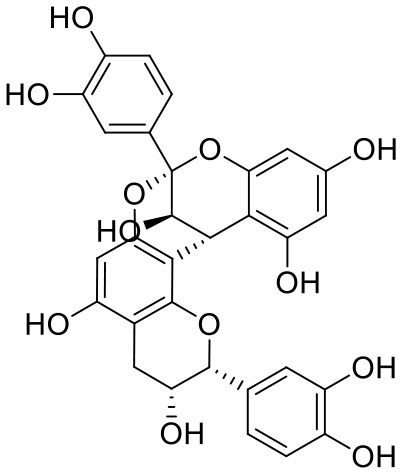	−7.302	−59.071
36	Calystegine C1	385737	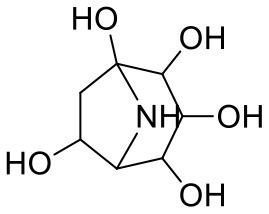	−7.234	−46.486
37	Chrysin	5281607	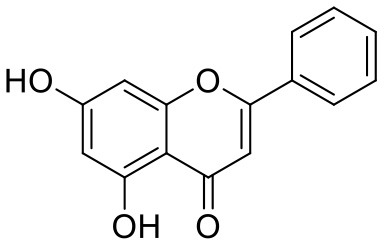	−7.162	−48.230
38	Genistein	5280961	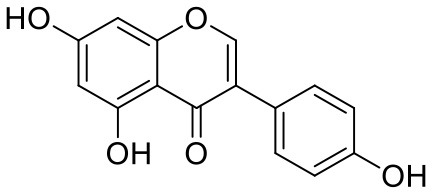	−7.138	−52.315
39	Arbutin	440936	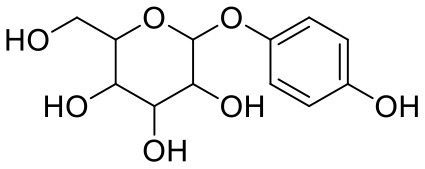	−7.137	−43.350
40	Apigenin	5280443	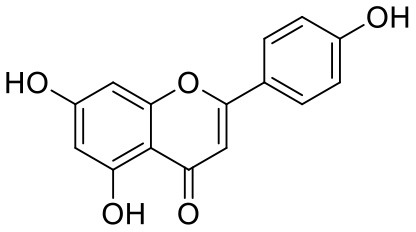	−7.090	−52.242
41	Curcumin	969516	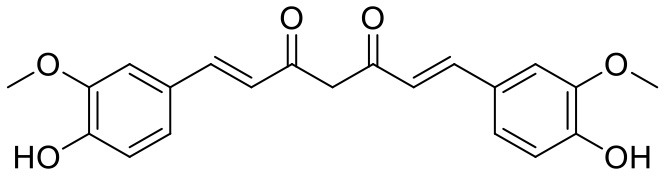	−7.077	−62.444
42	Luteoline	5280445	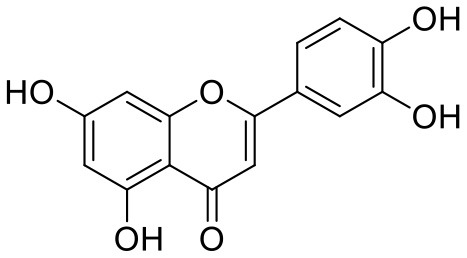	−7.071	−56.330
43	Robinetin	5281692	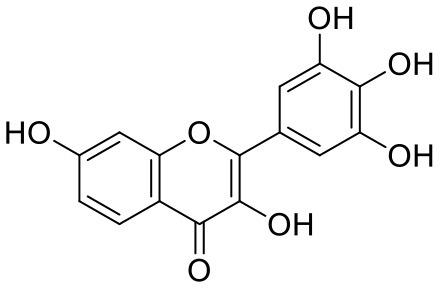	−7.057	−60.290
44	Theaflavin-3,3′-digallate	135403795	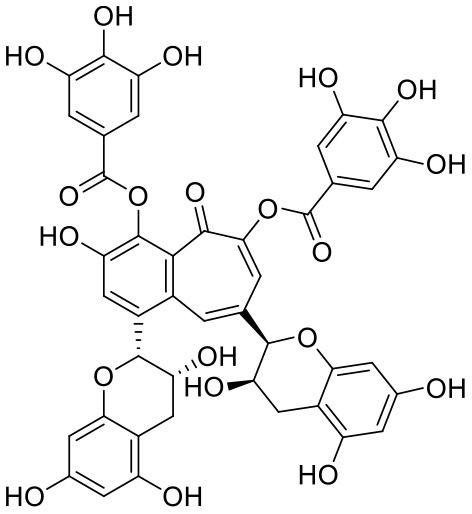	−7.010	−117.336
45	Ipolamiide	442425	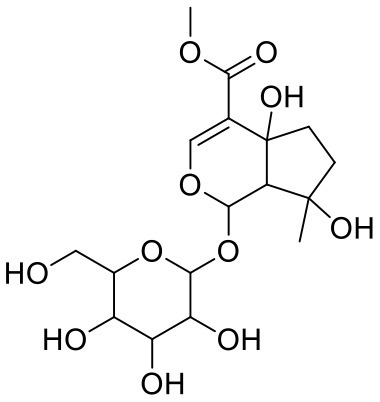	−6.996	−45.556
46	(-)-Gallocatechin gallate	199472	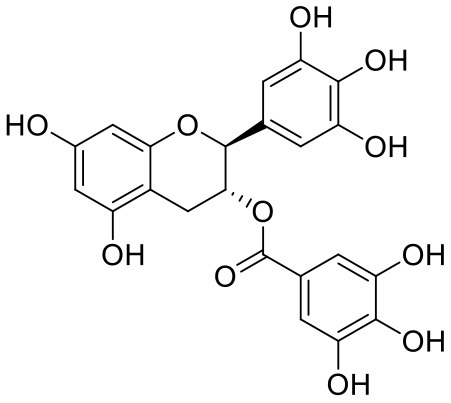	−6.847	−86.726
47	(-)-Catechin	73160	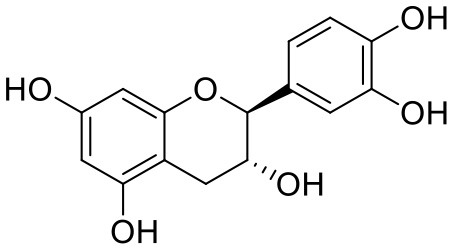	−6.709	−51.988
48	Mangiferin	5281647	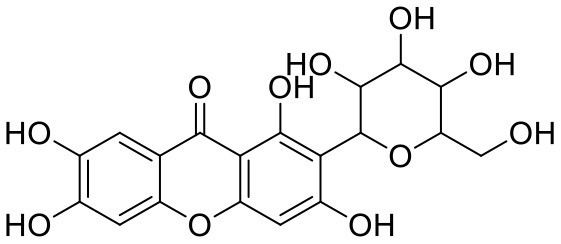	−6.651	−63.973
49	Rhamnetin	5281691	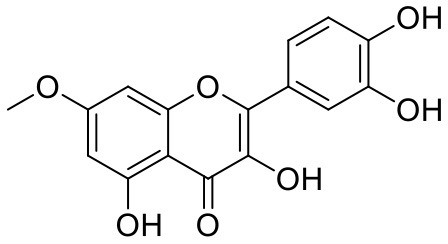	−6.578	−55.659
50	Cyanidin	128861	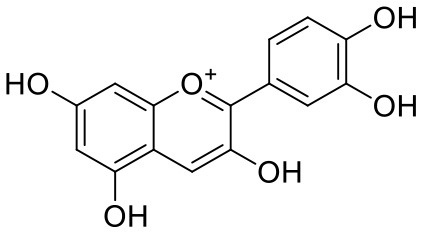	−6.535	−63.976
51	Epitheaflavin monogallate	135625500	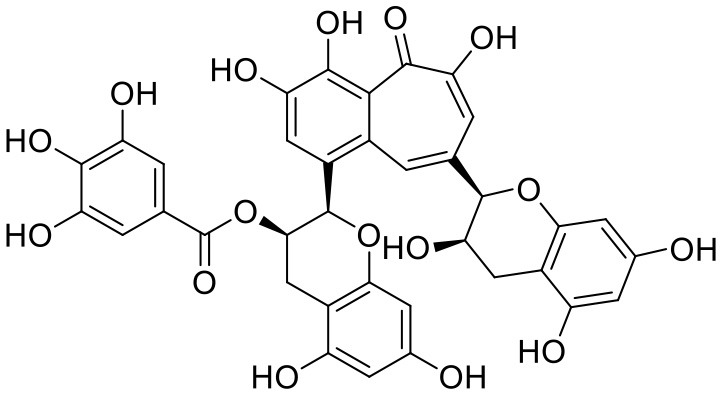	−6.505	−109.923
52	Cordifoliside A	101676711	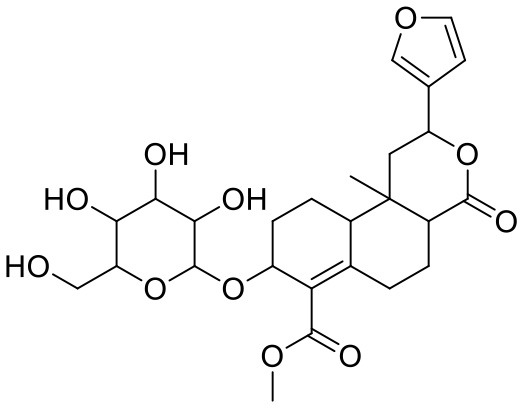	−6.428	−71.467
53	Ellagic acid	5281855	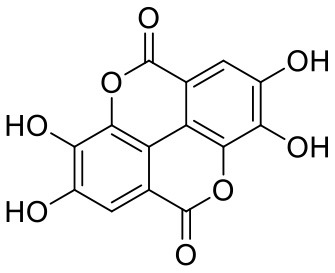	−6.393	−48.814
54	Calanolide	1201	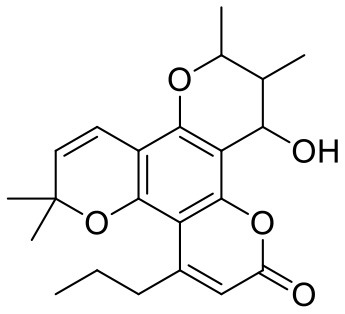	−6.391	−50.372
55	Morelloflavone	5464454	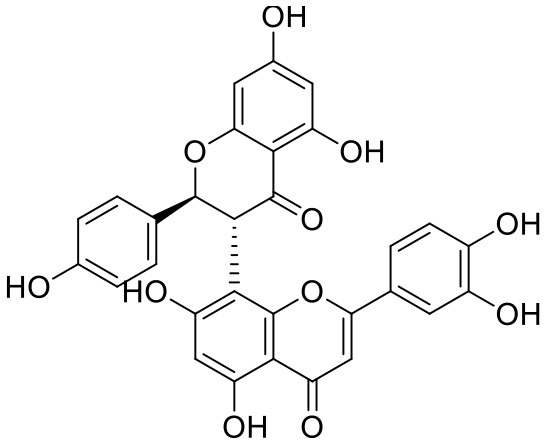	−6.300	−72.764
56	Aloe emodin	10207	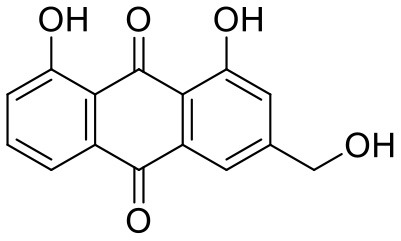	−6.229	−46.278
57	Scutellarin	185617	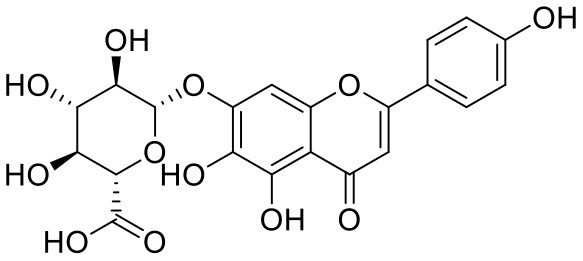	−6.137	−69.299
58	Cordifoliside C	101676208	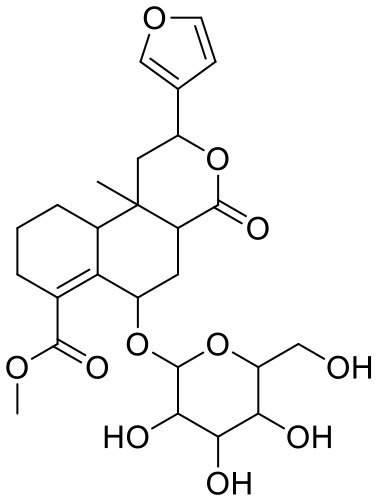	−6.123	−59.081
59	Cordifoliside B	101676207	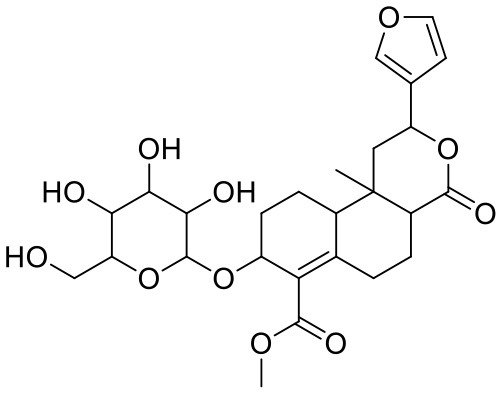	−5.965	−58.814
60	N3 (Co-crystal ligand)	-	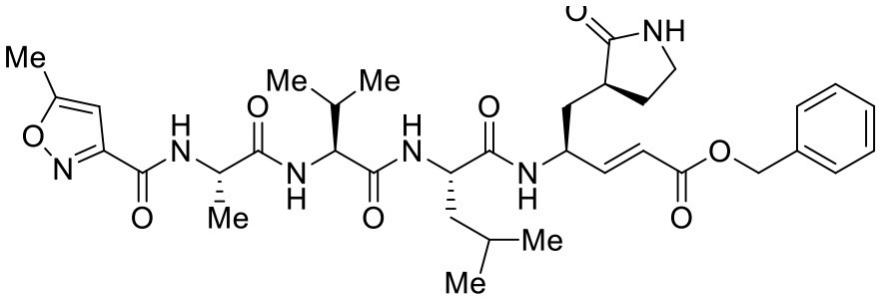	−7.93	−118.489

*Bold values show the top docking scores*.

To validate the generated grid for docking, the co-crystallized ligand (N3) of PDB 6LU7 was first knocked out, reconstructed, and re-docked into the active site of the receptor using the generated grid. Here, the N3 molecule showed a similar pattern of orientation and interactions such as hydrogen bonding with Glu166, Gln189, and Thr190 residues of the active site. The XP docking score between N3 and Mpro protein was −7.93 kcal/mol. This docking was analyzed further by recognizing the all-atom RMSD value of the re-docked N3 ligand with the co-crystallized ligand, and it was found to be 0.095 Å, which validated the docking protocol.

**Figure 2:**
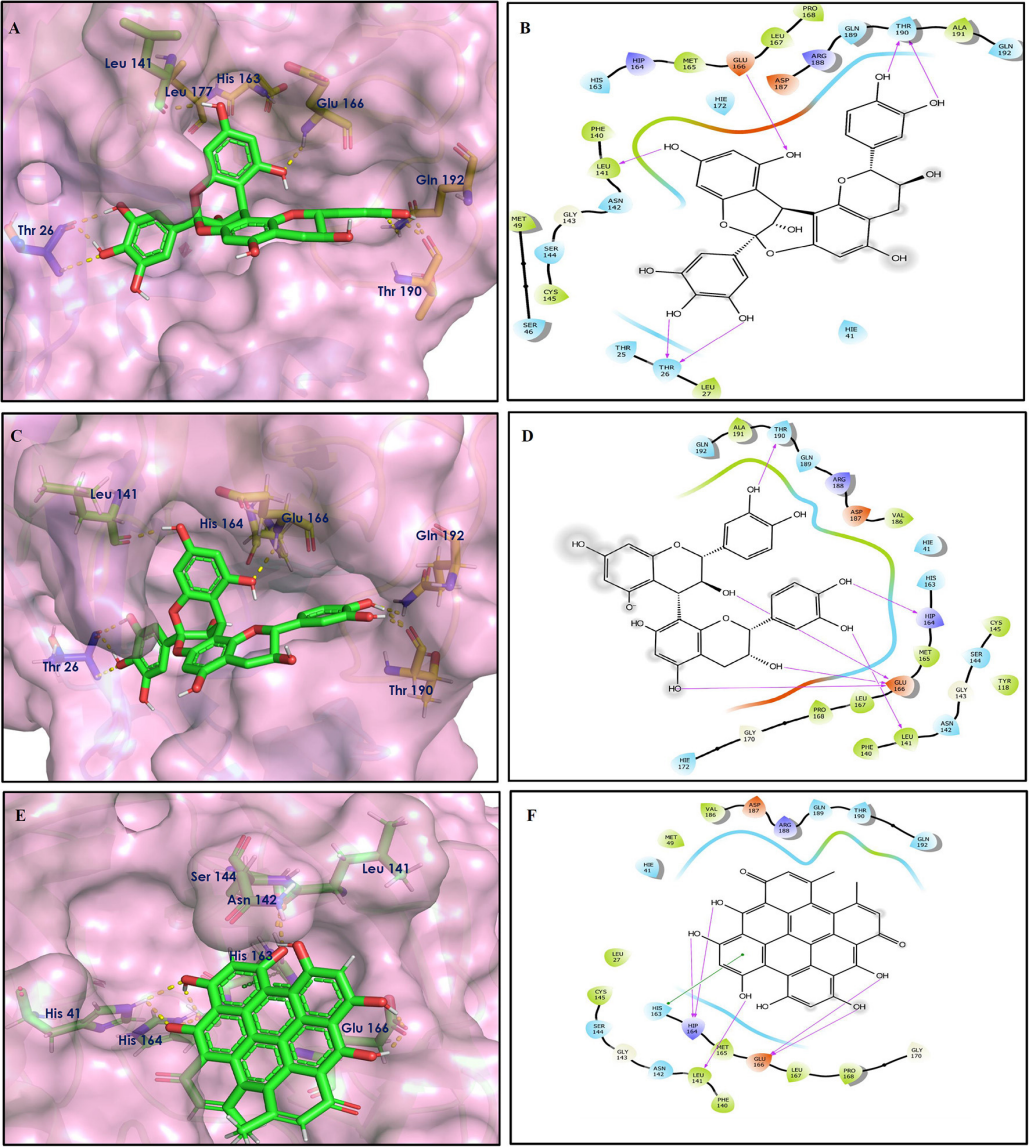
Docking interactions of compounds **(1, 3, 4**: **A–F)** in the active sites of Mpro. **(A,C,E)** 3D-binding mode of compounds **1**, **3**, and **4** with Mpro active site, respectively. Ligands are shown as green sticks, Mpro residues are shown as atom type color sticks, hydrogen bonds formed between ligands and receptor are depicted as yellow dotted lines. **(B,D,F)** 2D-ligand interaction diagram of compounds **1**, **3**, and **4** with Mpro active site, respectively.

**Figure 3:**
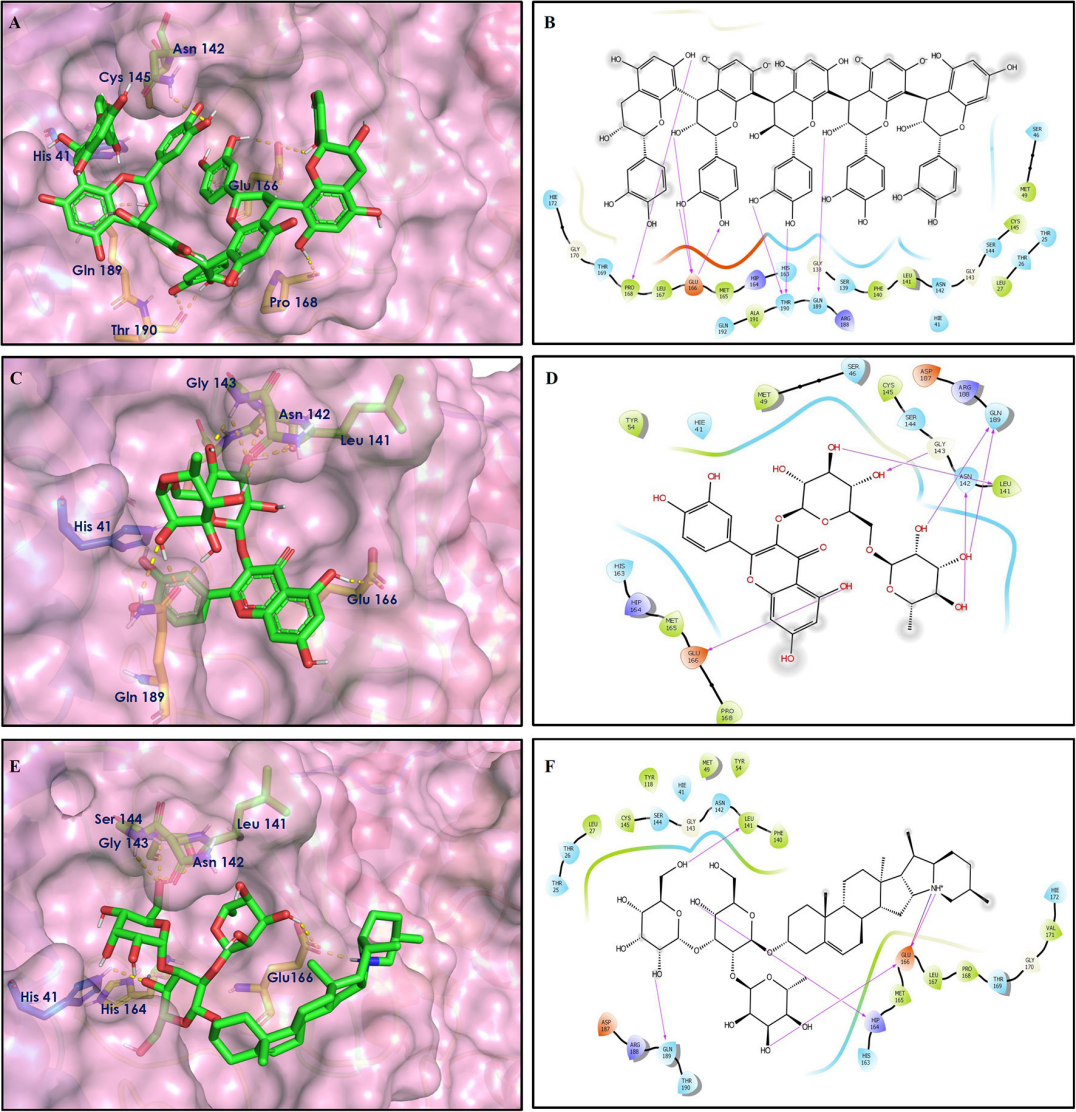
Docking interactions of compounds **(5, 6, 7**: **A–F)** in the active sites of Mpro. **(A,C,E)** 3D-binding mode of compounds **5**, **6**, and **7** with Mpro active site, respectively. Ligands are shown as green sticks, Mpro residues are shown as atom type color sticks, hydrogen bonds formed between ligands and receptor are depicted as yellow dotted lines, π-π stacking interaction is indicated as green dotted lines. **(B,D,F)** 2D-ligand interaction diagram of compounds **5**, **6**, and **7** with Mpro active site, respectively.

**Figure 4:**
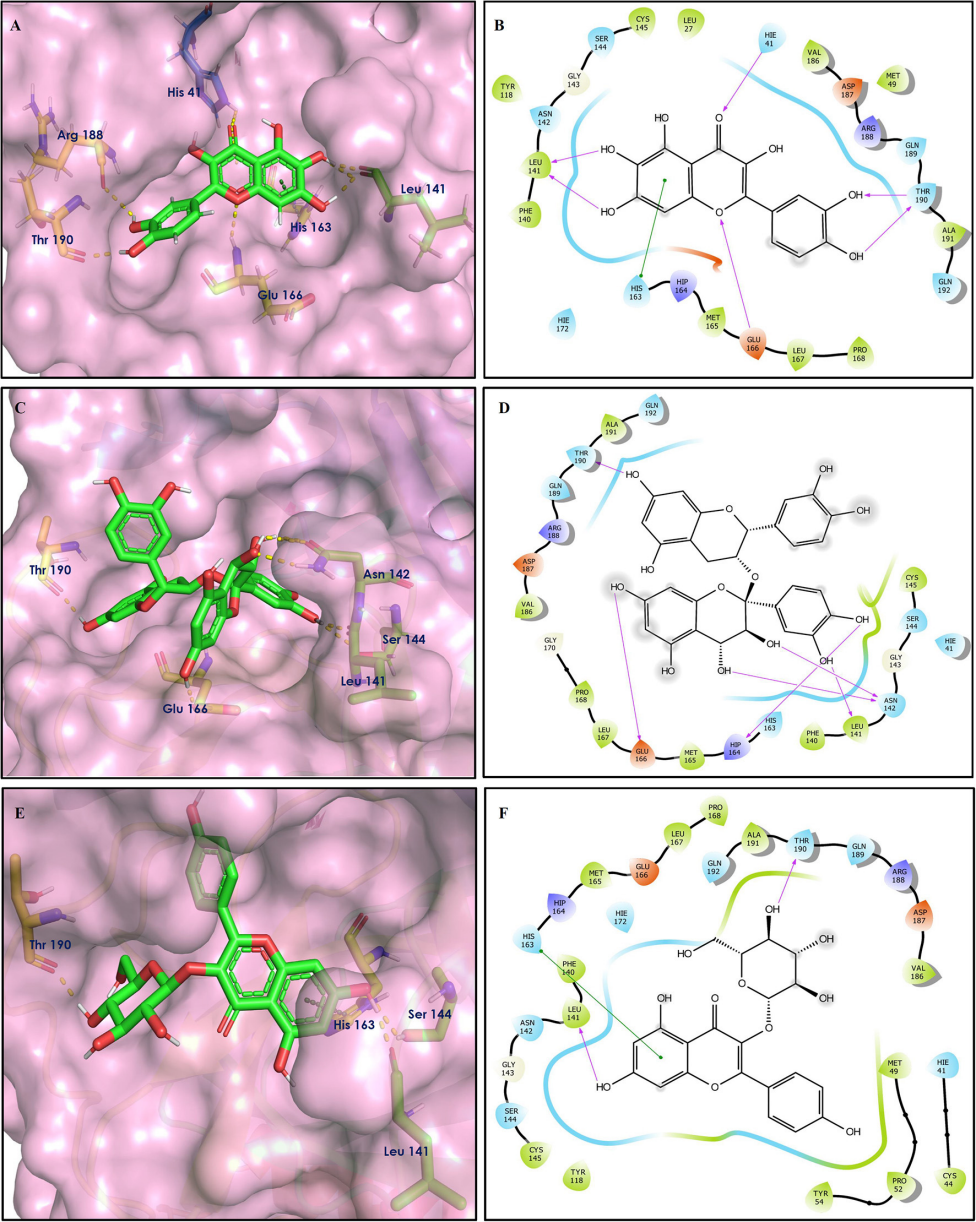
Docking interactions of compounds **(8, 9, 10**: **A–F)** in the active sites of Mpro. **(A,C,E)** 3D-binding mode of compounds **8**, **9**, and **10** with Mpro active site, respectively. Ligands are shown as green sticks, Mpro residues are shown as atom type color sticks, hydrogen bonds formed between ligands and receptor are depicted as yellow dotted lines, π-π stacking interaction is indicated as green dotted lines; **(B,D,F)** 2D-ligand interaction diagram of compounds **8**, **9**, and **10** with Mpro active site, respectively.

The top nine docked compounds, Procyanidin A3 (1), Rutin (3), Solanine (4), Procyanidin A4 (5), Procyanidin B4 (6), Hypericin (7), Quercetagetin (8), Procyanidin (9), and Astragalin (10) were selected for discussion in detail. Acetoside (2) possessing α, β-unsaturated carbonyl group as warhead is able to bind covalently with Cys145 residue of Mpro. The results obtained from covalent docking will be discussed in the *Covalent Docking* section. The ligand–receptor interaction diagrams are shown in Figures 2–4.

Procyanidins are oligomeric catechin or epicatechin having significant medicinal values. Procyanidin A3 (1) is pentamer of epicatechin (Park et al., 2014). It showed the highest docking score (−12.86 kcal/mol) against Mpro of SARS CoV-2 in the present study. Hydroxyl groups of procyanidin A3 showed H-bonding with Pro168 (2.24 Å), Glu166 (1.68 Å, 1.98 Å, 2.77 Å), Thr190 (1.86 Å, 1.92 Å), and Gln189 (2.03 Å) amino acid residues.

Rutin (3) is a bio-flavonoid glycoside found in many plants including *Fagopyrum esculantum, Eucalyptus Sps, Ruta graveolens*, and *Tephrosia purpurea*. Chemically, it is a glycoside comprising of flavonol aglycone quercetin along with disaccharide rutinose. It is found to have a number of pharmacological activities, including antioxidant, cytoprotective, vasoprotective, anticarcinogenic, neuroprotective, and cardioprotective activities (Enogieru et al., 2018). One of the hydroxyl groups of chromane ring of rutin showed H-bonding with Glu166 (2.16 Å). Hydroxyl groups of L-rhamnopyranose of rutin showed H-bonding with Gln189 (1.85 Å, 1.86 Å) and Asn142 (1.96 Å), and D-glucopyranose showed H-bonding with Leu141 (1.86 Å) and Gly143 (2.07 Å).

Solanine (4) is a glycoalkaloid of the genus *Solanum*, such as the European black nightshade (*Solanum nigrum*), potato (*Solanum tuberosum*), tomato (*Solanum lycopersicum*), and eggplant (*Solanum melongena*). It has fungicidal, antimicrobial, and pesticidal properties (Zhao et al., 2018). The nitrogen atom of solanine that got protonated at physiological pH showed salt bridge with Glu166 (4.87 Å) and H-bonding with Glu166 (1.93 Å). The hydroxyl group of D-glucopyranose attached to the steroidal backbone showed H-bonding with His164 (1.76 Å). The next D-glucopyranose sugar also interacted by H-bonding of the methylene hydroxyl group with Leu141 (1.90 Å) and H-bonding of the hydroxyl group with Gln189 (2.78 Å). The hydroxyl group of L-rhamnopyranose formed H-bonding with Glu166 (2.64 Å).

Procyanidin A4 (5) is also having multiple aromatic hydroxyl groups, which form hydrogen bonds with amino acid residues of the target protein. Two aromatic hydroxyl groups formed hydrogen bonds with Thr26 at a distance of 1.79 and 1.94 Å. Another pair of the hydroxyl groups also showed H-bonding with Thr190 (1.84, 1.99 Å). Another two hydroxyl groups of chromane ring showed H-bonding with Glu 166 (2.24 Å) and Leu141 (2.04 Å).

Procyanidin B4 (6) is a catechin-(4α → 8)-epicatechin dimer. It is found in the litchi pericarp, in grape seeds, and, along with 4-cis-isomer of procyanidin B4, in beer. It has a role as an antioxidant, a DNA topoisomerase (ATP-hydrolyzing) inhibitor, and an antineoplastic agent (Zhao et al., 2007). Multiple hydroxyl groups present in the structure showed H-bonding with Glu166 (1.77, 2.21, 2.27 Å), Leu141 (1.81 Å), His164 (2.06 Å), and Thr190 (1.69 Å) residues.

Hypericin (7) is a naphthodianthrone, an anthraquinone derivative, which is found in the flower of *Hypericum perforatum* (St. John's wort). It is found to have antidepressant, potential antiviral, antineoplastic, and immunomodulating activities (Vollmer and Rosenson, 2004). The aromatic phenyl ring allowed the creation of a more favorable π-π stacking with His163 (5.49 Å). The hydroxyl groups present in hypericin showed H-bonding with Glu166 (1.99, 2.06 Å), Leu141 (1.74 Å), and His164 (2.13, 2.14 Å).

Quercetagetin (8) is a hexahydroxy flavone, found in *Citrus unshiu* abundantly. It has antioxidant and antiviral properties (Kang et al., 2013). Phenolic hydroxyl groups of the chromone ring showed H-bonding with Leu141 (1.67, 1.93 Å). The oxygen of the chromone ring showed H-bonding with Glu166 (2.40 Å), and the carbonyl group oxygen of chromone showed H-bonding with His41 (2.61 Å). The aromatic phenyl ring of chromone showed π-π stacking with His163 (5.30 Å). Phenolic hydroxyl groups attached to the phenyl ring at C-2 position of the chromone ring showed H-bonding with Thr190 (2.10, 2.76 Å).

Procyanidin (9) contains multiple hydroxyl groups, which showed H-bonding with Glu166 (1.69 Å), His164 (1.80 Å), Leu141 (1.88 Å), Asn142 (1.96, 2.48 Å), and Thr190 (1.97 Å) residues.

Astragalin (10) is a flavonoid, 3-O-glucoside of kaempferol, found in plants such as *Allium ursinum, Allium sativum, Cassia alata, Cuscuta chinensis*, and *Phytolacca americana*. It is having diversified pharmacological activities such as anti-inflammatory, antioxidant, neuroprotective, cardioprotective, antiobesity, antiosteoporotic, anticancer, antiulcer, and antidiabetic (Riaz et al., 2018). The phenolic hydroxyl group of the chromone ring showed H-bonding with Leu141 (2.00 Å). The aromatic phenyl ring of chromone showed π-π stacking with His163 (5.18 Å). The hydroxyl group of glucopyranose formed H-bonding with Thr190 (1.99 Å).

#### Covalent Docking

**Figure 5:**
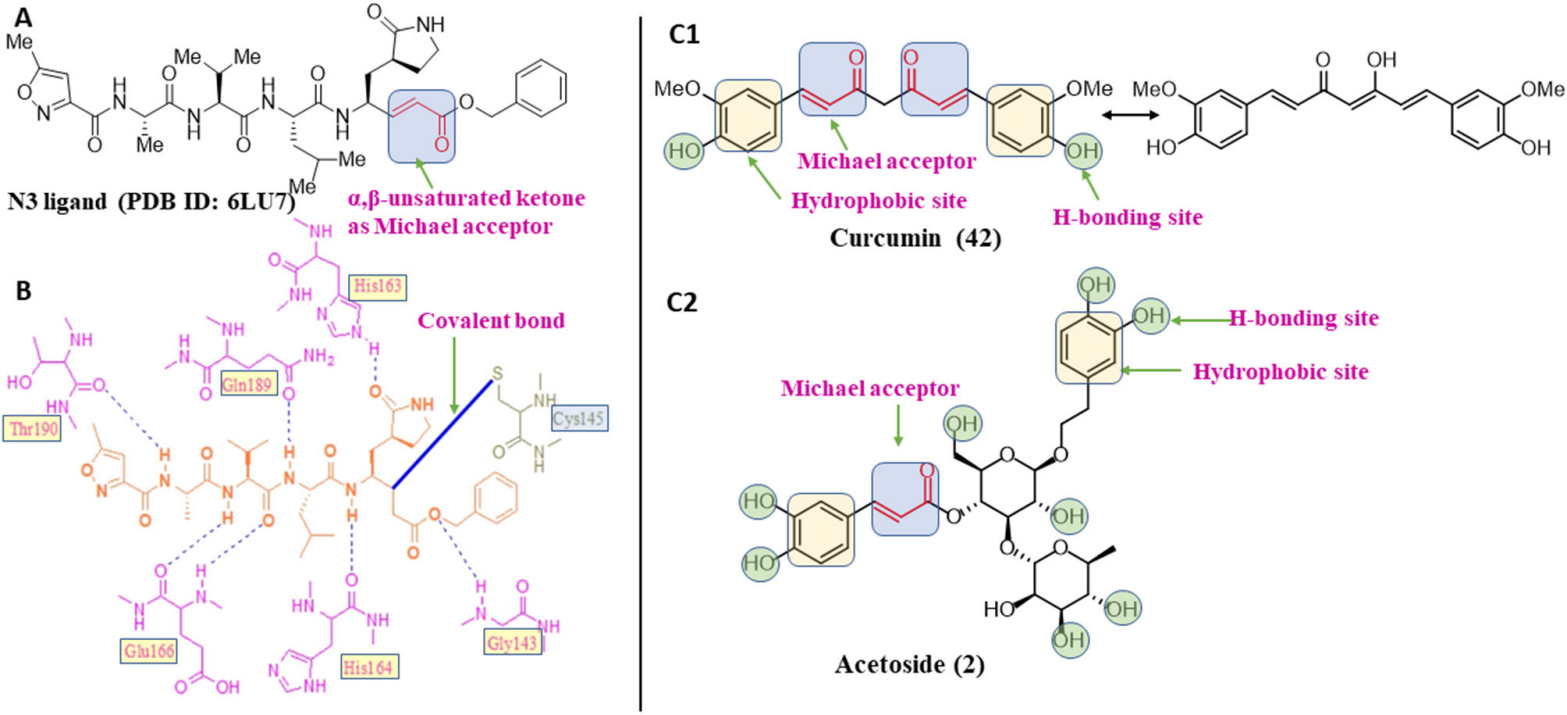
Structural characteristics of N3 ligand of SAR-CoV-2 Mpro (PDB ID: 6LU7). **(A)** Important structural feature offered by N3 ligand as covalent inhibitor. **(B)** 2D-interaction diagram of ligand N3 and Mpro, dark blue colored solid line depicted covalent bond (Jin et al., 2020); **(C1)** Important structural features offered by the curcumin as covalent inhibitor; **(C2)** Important structural features of acetoside as covalent inhibitor.

Covalent inhibitors have a prolonged history in drug discovery, beginning in the late nineteenth century with aspirin and continuing with a current surge of rationally designed kinase inhibitors enrolling in clinical trials (Ghosh et al., 2019). N3 ligand has shown covalent bonding with Cys145 of Mpro with −7.466 kcal/mol docking score in the current study. The literature demonstrated that Cys145 is a key residue in the active site of Mpro, which is found to be an attractive target for covalent modification by Mpro inhibitors. Cys145 amino acid residue in the active site of Mpro covalently attaches to the β-position of the peptide-like α, β-unsaturated carbonyl ligand N3 by Michael addition reaction (Jin et al., 2020) (Figures 5A,B). In addition to this, it was also noted that the ligand N3 has adequate hydrogen bonding and stacking interactions with different hydrophilic and hydrophobic regions of the protein. These structural features like α, β-unsaturated ketone, hydrophilic, and hydrophobic regions are also found in the structures of acetoside (2) and curcumin (42) (Figures 5C1,C2). The distance between the sulfur atom of Cys145 and the covalent carbon atom of N3 ligand is 1.8 Å. This distance is around the same for acetoside (2) and curcumin (42).

**Figure 6:**
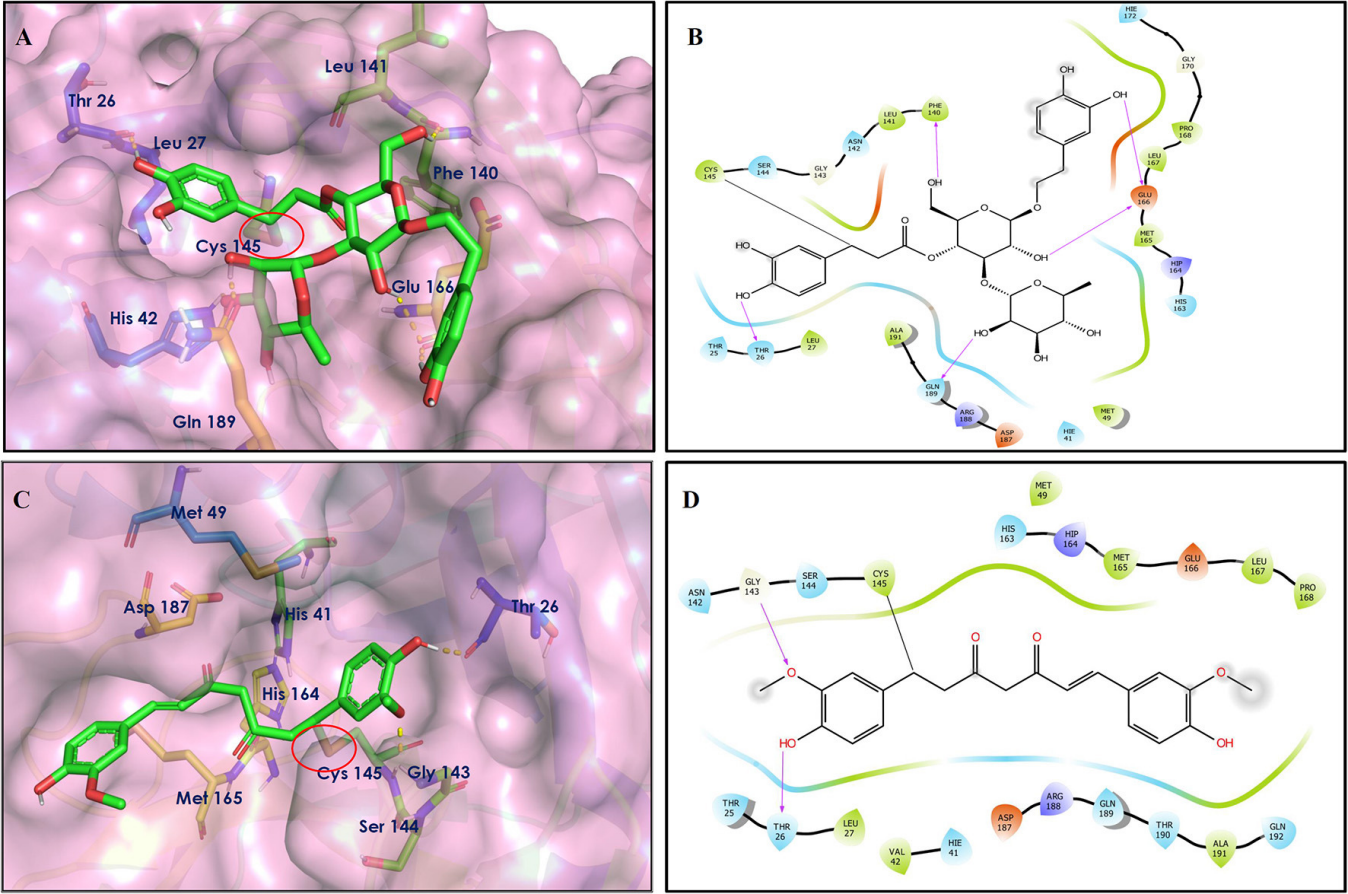
Covalent docking interactions of compounds **(2, 42: A–D)** in the active sites of Mpro. **(A,C)** 3D-Binding mode of compounds **2** and **42** with Mpro active site, respectively. Ligands are shown as green sticks, Mpro residues are shown as atom type color sticks, hydrogen bonds formed between ligands and receptor are depicted as yellow dotted lines, the red circle highlights the C–S covalent bond. **(B,D)** 2D-ligand interaction diagram of compounds **2** and **42** with Mpro active site, respectively.

The covalent binding modes of acetoside and curcumin are described in Figure 6.

Leaves and drupes of olive tree, *Olea europaea* and *Verbascum phlomoides* are rich in acetoside (2). It has shown antioxidant, antibacterial neuroprotectivity, and anti-inflammatory activity. Acetoside is a glycoside that is the α-L-rhamnosyl-(1→ 3)-β-D-glucoside of hydroxytyrosol (Shiao et al., 2017). Acetoside is able to fit snugly inside the Mpro with covalent binding score −6.91 kcal/mol. The docked pose analysis of acetoside revealed that it formed five H-bonds with amino acid residues Thr26 (1.76 Å), Phe140 (2.28 Å), Glu166 (1.82, 2.21 Å), and Gln189 (1.74 Å) present at the active site of Mpro. The α, β-unsaturated ketone in the structure showed a covalent bond interaction (1.84 Å) between the β-position of ketone and Cys145 residue. The hydroxyl groups of acetoside showed H-bonding with Thr26 (1.99 Å) and Leu141 (1.76 Å, 2.27 Å). The keto group of acetoside showed H-bonding with His41 (2.13 Å).

The medicinal value of curcumin (42) (Turmeric) has been known since ancient times in India. It belongs to the *Zingiberaceae* family and is found in the rhizome of *Curcuma longa* and other *Curcuma* species (Hewlings and Kalman, 2017). Curcumin is also able to covalently dock inside the Mpro with a covalent binding score of −7.028 kcal/mol. The phenolic hydroxyl group showed H-bonding with Thr26 residue (1.66 Å), and the oxygen of the methoxy group on phenyl ring showed H-bonding with Gly143 residue (2.10 Å). Curcumin is found to be involved in an important covalent interaction with Cys145 residue of Mpro through Michael addition. The α, β-unsaturated ketone in curcumin showed a covalent bond (1.83 Å) between the β-position of ketone and Cys145 residue.

#### Spike Glycoprotein Docking

**Figure 7:**
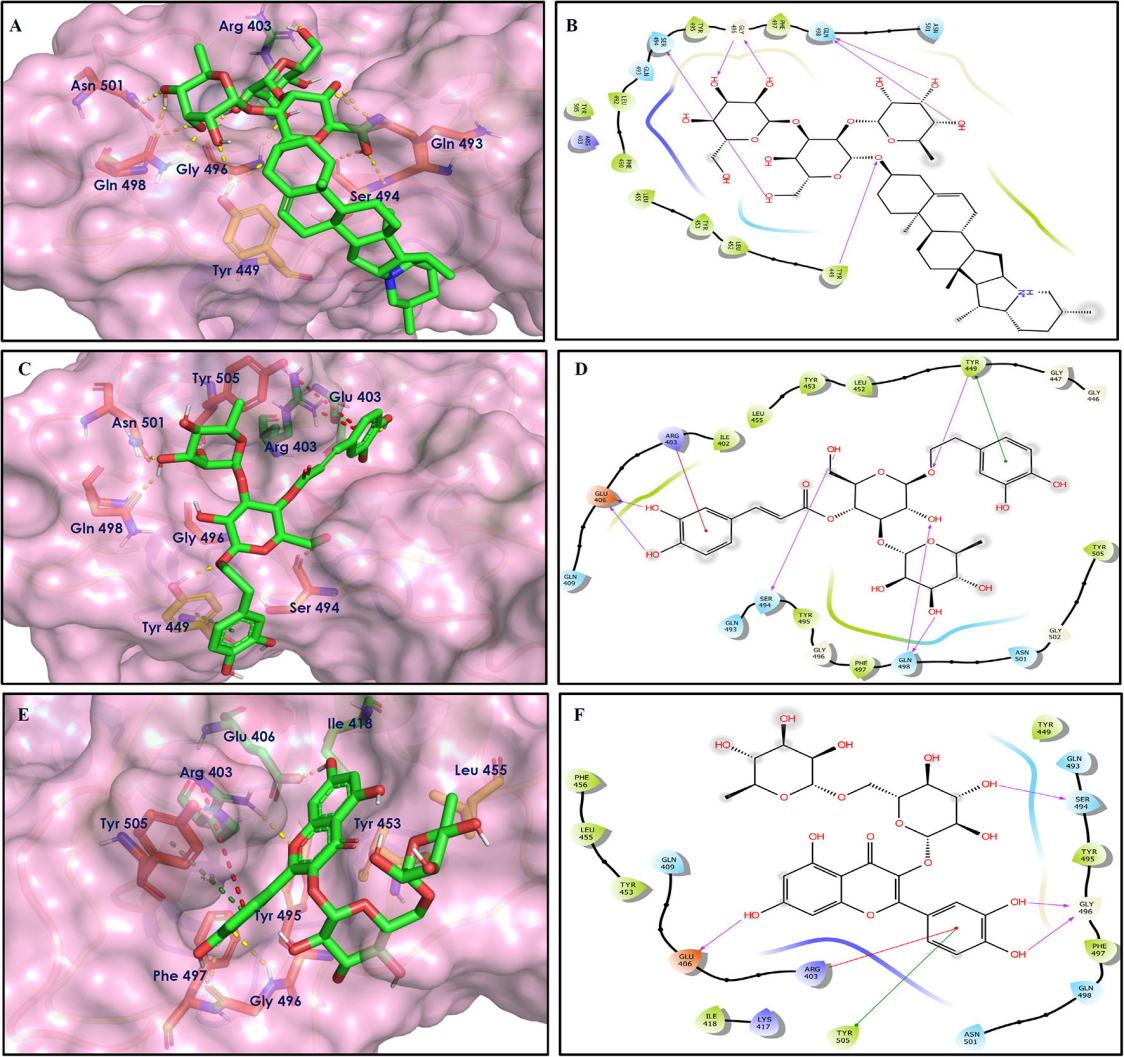
Docking interactions of compounds **(4, 2, 3**: **A–F)** in the active sites of Spike RBD. **(A,C,E)** 3D-binding mode of compounds **4**, **2**, and **3** with Spike RBD active site, respectively. Ligands are shown as green sticks, Spike RBD residues are shown as atom type color sticks, hydrogen bonds formed between ligands and receptor are depicted as yellow dotted lines, π-π stacking interaction as green dotted lines and π-cation interaction as red dotted lines. **(B,D,F)** 2D-ligand interaction diagram of compounds **4**, **2**, and **3** with Spike RBD active site, respectively.

**Figure 8:**
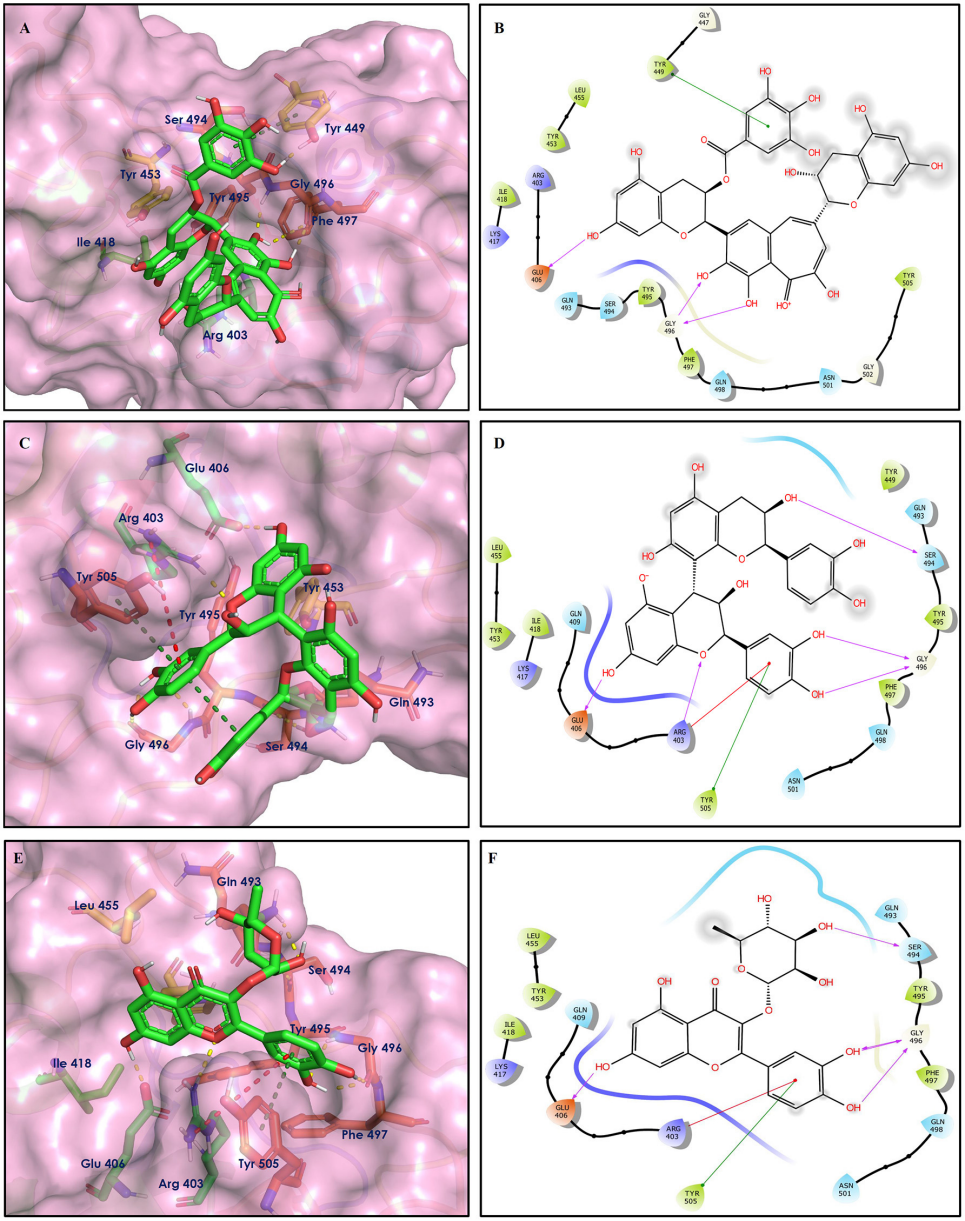
Docking interactions of compounds **(52, 14, 19**: **A–F)** in the active sites of Spike RBD. **(A,C,E)** 3D-binding mode of compounds **52**, **14**, and **19** with Spike RBD active site, respectively. Ligands are shown as green sticks, Spike RBD residues are shown as atom type color sticks, hydrogen bonds formed between ligands and receptor are depicted as yellow dotted lines, π-π stacking interaction as green dotted lines and π-cation interaction as red dotted lines. **(B,D,F)** 2D-ligand interaction diagram of compounds **52**, **14**, and **19** with Spike RBD active site, respectively.

**Figure 9:**
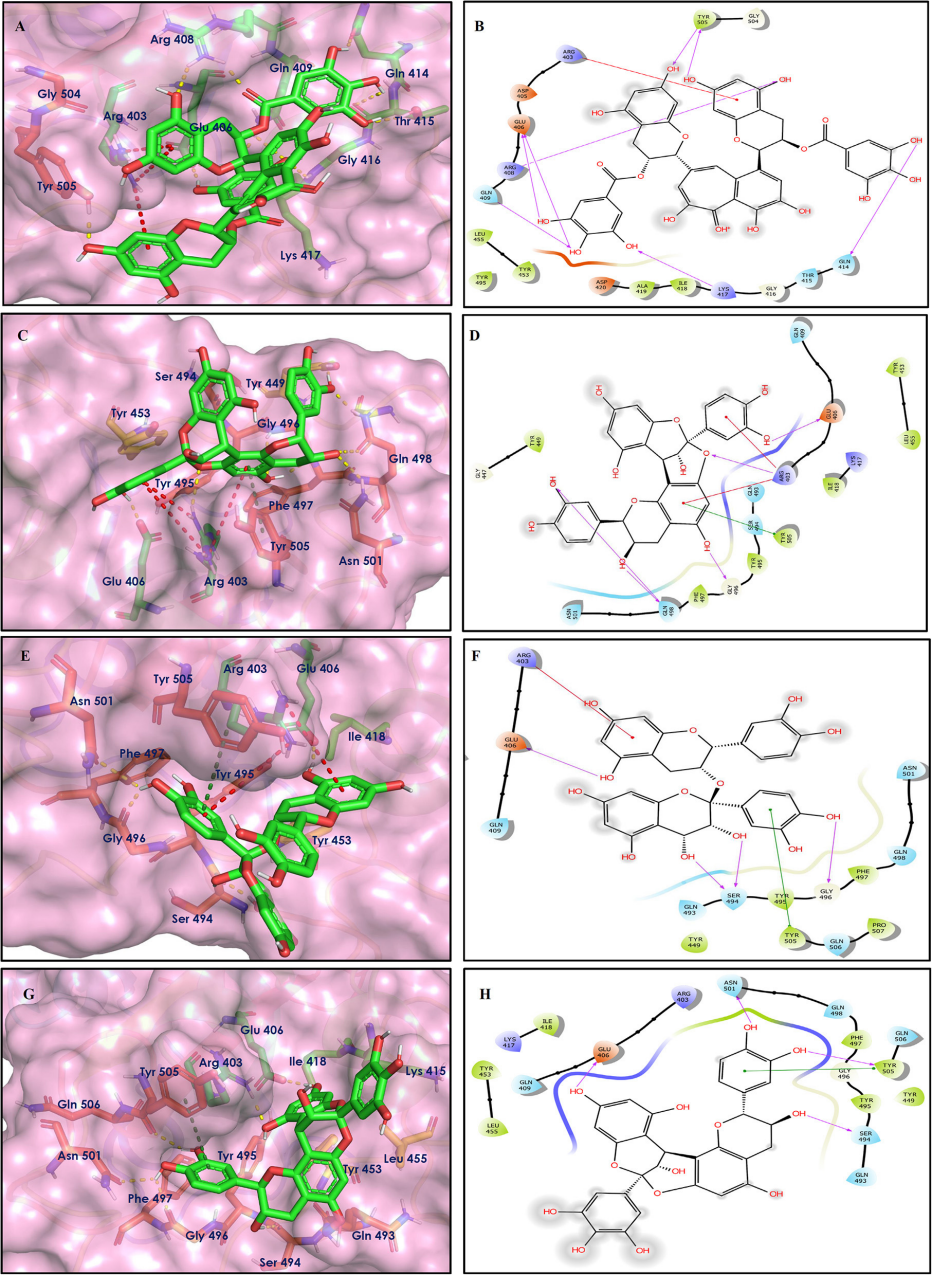
Docking interactions of compounds **(45, 11, 9, 5**: **A–H)** in the active sites of Spike RBD. **(A,C,E,G)** 3D-binding mode of compounds **45**, **11, 9**, and **5** with Spike RBD active site, respectively. Ligands are shown as green sticks, Spike RBD residues are shown as atom type color sticks, hydrogen bonds formed between ligands and receptor are depicted as yellow dotted lines, π-π stacking interaction as green dotted lines and π-cation interaction as red dotted lines. **(B,D,F,G)** 2D-ligand interaction diagram of compounds **45**, **11, 9**, and **5** with Spike RBD active site, respectively.

To study the Spike protein RBD bound to ACE2 interactions, the same 170 phytoconstituents were also subjected to molecular docking against the receptor-binding domain (RBD) of the S-protein (PDB ID: 6M0J). The top 10 docked compounds are shown in Table 2. Their 2D and 3D interactions are described below in Figures 7–9.

**Table 2:** Chemical structures and docking results of phytoconstituents on spike receptor-binding domain of SARS CoV-2 bound to the ACE2.

**Comp. No**.	**Name**	**PubChem ID**	**Docking score** **against spike glycoprotein (kcal/mol) (PDB ID: 6M0J)**	**Glide emodel (kcal/mol)**
**4**	Solanine	262500	−9.501	−65.597
**2**	Acetoside	5281800	−8.528	−65.024
**3**	Rutin	5280805	−7.911	−63.472
**52**	Epitheaflavin monogallate	620853	−7.524	−70.219
**14**	Procyanidin B2	122738	−7.428	−65.827
**19**	Quercitrin	5280459	−7.152	−60.829
**45**	Theaflavin 3,3′-digallate	135403795	−7.027	−82.725
**11**	Procyanidin A1	5089889	−6.836	−66.553
**9**	Procyanidin	107876	−6.768	−61.772
**5**	Procyanidin A4	53349182	−6.690	−70.376

Solanine (4) showed good binding affinity with spike glycoprotein RBD also similar with Mpro and exhibited −9.501 kcal/mol docking score against PDB ID 6M0J. The oxygen atom of glycosidic bond, attached with a steroidal backbone participated in the H-bonding with Tyr449 (2.64 Å). The methylene hydroxyl group of glucopyranose, attached with a steroidal backbone showed H-bonding with Ser494 (1.92 Å). The hydroxyl groups of the next glucopyranose sugar showed H-bonding with Gly496 (2.14, 2.18 Å). The hydroxyl groups of rhamnopyranose exhibited H-bonding with Gln498 (2.14, 2.15 Å).

Acetoside (2) also showed remarkable binding affinity with spike glycoprotein RBD (−8.528 kcal/mol). Hydroxyl groups attached to the phenyl ring at the β-position of the carbonyl group showed H-bonding with Glu406 (1.53, 2.51 Å). This phenyl ring showed strong π-cation interaction with Arg403 (5.27 Å). The other phenyl ring showed π-π stacking with Tyr449 (3.93 Å). The oxygen atom of ethoxy chain was found to have H-bonding with Tyr449 (1.96 Å). The hydroxyl group of rhamnopyranose exhibited H-bonding with Gln498 (1.98 Å). The hydroxyl group of glucopyranose sugar also showed H-bonding with Gly498 (2.79 Å). The methylenehydroxyl group of glucopyranose sugar showed H-bonding with Ser494 (1.98 Å).

Rutin (3) showed a −7.911 kcal/mol docking score against spike glycoprotein RBD. One of the hydroxyl groups of the chromane ring of rutin showed H-bonding with Glu406 (1.79 Å). The hydroxyl group of glucopyranose of rutin showed H-bonding with Ser494 (1.93 Å). The hydroxyl groups attached to the phenyl ring at the C-2 position of the chromone ring showed H-bonding with Gly496 (1.71, 2.03 Å). This phenyl ring showed strong cation-π interaction with Arg403 (6.34 Å) and π-π stacking with Tyr505 (4.75 Å).

Epitheaflavin monogallate (52) is one of the major polyphenols of black tea (Łuczaj and Skrzydlewska, 2005). It exhibited −7.524 kcal/mol docking score against spike glycoprotein RBD. Two hydroxyl groups attached to the benzotropolone ring showed H-bonding with Gly496 (1.70, 2.07 Å). The hydroxyl group of the chromane ring showed H-bonding with Glu406 (1.85 Å). The terminal trihydroxyphenyl (gallate) ring showed a strong π-π interaction with Tyr449 (5.48 Å).

Procyanidin B2 (14) showed a −7.428 kcal/mol docking score against spike glycoprotein RBD. The hydroxyl groups attached to the basic flavone rings showed H-bonding with Glu406 (1.85 Å), Ser484 (2.21 Å), and Gly496 (2.01, 2.21 Å) residues. Dihydroxyphenyl ring at C2 position of dihydroxychromone showed strong π-cation interaction with Arg403 (6.42 Å) and π-π stacking with Tyr505 (4.83 Å). The oxygen atom of dihydroxychromone ring showed H-bonding with Arg403 (1.96 Å).

Quercitrin (19), a quercetin 3-rhamnoside possessed relatively better binding affinity with spike glycoprotein RBD. The hydroxyl group at the C-7 position of the 5,7-dihydroxychromone ring showed H-bonding with Glu406 (1.66 Å). The two hydroxyl groups of the catechol ring showed H-bonding with Gly496 (2.10, 2.51 Å). This catechol ring provides additional stability to the ligand–receptor complex by forming strong π-cation interaction with Arg403 (6.39 Å) and π-π stacking with Tyr505 (4.80 Å). The hydroxyl group at the C-5 position of rhamnose sugar formed an H-bond with Ser494 (2.40 Å).

Theaflavin 3,3′-digallate (45) is an antioxidant natural polyphenol found in black tea (Łuczaj and Skrzydlewska, 2005). It interacted through several H-bonds with amino acid residues of 6M0J. The hydroxyl groups at C-7 positions of 5,7-dihydroxychromane ring showed H-bonding with Tyr505 (2.12, 2.05 Å). The hydroxyl group at C-5 positions of 5,7-dihydroxychromane ring showed H-bonding with Arg408 (2.25 Å). This 5,7-dihydroxychromane ring provides additional stability to the ligand–receptor complex by forming π-cation interaction with Arg403 (6.01 Å). The terminal trihydroxyphenyl (gallate) ring showed H-bonding interaction with hydroxyl groups and with Glu406 (1.88, 2.01 Å), Gln409 (2.06 Å), Lys417 (2.17 Å), and Gln414 (2.05 Å) residues.

In Procyanidin A1 (11), the stability to the ligand–receptor complex is mainly provided by the hydroxyl groups present in the structure by forming multiple H-bonding with various amino acid residues [i.e., Glu406 (1.80 Å), Gly496 (2.08 Å), and Gln498 (2.24, 1.98 Å)]. One oxygen atom of the ring showed H-bonding with Arg403 (2.39). The aromatic rings present in the structure provided the additional stability to ligand receptor complex by forming strong π-cation interaction with Arg403 (5.78, 6.02 Å) and π-π stacking with Tyr505 (4.75 Å).

In Procyanidin (9), the hydroxyl groups at the C-3 and C-4 positions of 5,7-dihydroxychromane ring showed H-bonding with Ser494 (1.78, 1.84 Å). The hydroxyl group at the C-5 position of the 5,7-dihydroxychromane ring showed H-bonding with Glu406 (1.68 Å). This 5,7-dihydroxychromane ring provides additional stability to the ligand–receptor complex by forming a π-cation interaction with Arg403 (6.01 Å). The catechol ring present in the structure provides stability to the complex by forming π-π interaction with Tyr505 (5.45 Å) and H-bonding between the hydroxyl group and Gly496 (1.81 Å).

In Procyanidin A4 (5), the stability to the ligand–receptor complex is mainly provided by the hydroxyl groups present in the structure by forming multiple H-bonding with various amino acid residues [i.e., Glu406 (1.68 Å), Tyr505 (2.15 Å), Asn501 (2.13 Å), and Ser494 (1.84 Å)]. The catechol ring present provides additional stability to the ligand–receptor complex by forming a π-π stacking interaction with Tyr505 (5.44 Å).

**Figure 10:**
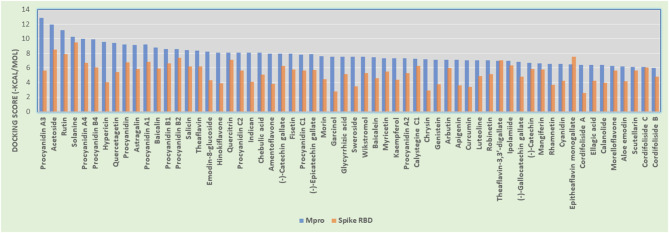
Histogram showing the comparison between docking scores of phytoconstituents (listed in Table 1) against Mpro and Spike RBD.

The phytoconstituents (listed in Table 1) that showed good Mpro inhibition *in silico* were also investigated in the same manner for their spike glycoprotein RBD inhibitory potential. From the above molecular docking studies against most promising targets of SARS-CoV-2 virus [i.e., Mpro and Spike RBD bound to ACE2, we found that phytoconstituents like Acetoside (2), Rutin (3), Solanine (4), Procyanidin A4, and Procyanidin (9) showed dual receptor inhibition, i.e., Mpro and Spike RBD inhibition]. The selected set of phytoconstituents are compared for their dual receptor inhibitory actions shown in Figure 10.

#### *In silico* Prediction of Physicochemical and Pharmacokinetics Parameters

The phytoconstituents that showed dual receptor inhibition, potent Mpro inhibition, and potent spike RBD inhibition *in silico* were further subjected for their *in silico* physicochemical and pharmacokinetic parameter prediction. The main purpose of these investigation is to afford “druglike” molecules. The most “druglike” molecules should have LogP ≤ 5, molecular weight ≤500, number of hydrogen bond acceptors ≤10, and number of hydrogen bond donors ≤5 according to Lipinski (Patel et al., 2019). The physicochemical parameters (Table 3) and pharmacokinetic profile indicators (Table 4) like volume, QPlogS, QPlogHERG, QPlogBB, QPPCaco, and percentage human oral absorption were predicted with QikProp module of Schrödinger.

**Table 3:** Predicted physicochemical parameters of phytoconstituents^a^.

**Sr. No**.	**Name**	**Source**	**Chemical Class**	**MW** **(g/mol)**	**HBD**	**HBA**	**QPlogP_**o/w**_**	**PSA**	**Rule of five (Violation)**
1	Procyanidin A3	*Vitis vinifera*	Flavonoid	1,443.29	25	27	−0.79	545.392	3
2	Acetoside	*Olea europaea, Verbascum phlomoides*	Polyphenol	624.59	9	20	−1.73	250.537	3
3	Rutin	*Fagopyrum esculentum, Eucalyptus Sps., Ruta graveolens*, *Tephrosia purpurea*	Flavonoid	610.52	9	20	−2.695	270.732	3
4	Solanine	*Solanum Sps*.	Glycoalkaloid	868.06	9	27	−0.221	220.726	3
5	Procyanidin A4	*Vitis vinifera*	Catechins	592.51	10	11	−0.057	230.429	3
6	Procyanidin B4	*Vitis vinifera*	Catechins	578.52	10	10	0.592	222.839	3
7	Hypericin	*Hypericum perforatum*	Dimeric anthraquinone	504.45	4	7	2.094	166.191	2
8	Quercetagetin	*Citrus unshiu*	Flavanoid	318.23	5	6	−0.208	160.06	1
9	Procyanidin	*Vitis vinifera*	Catechins	594.52	10	11	0.076	232.723	3
10	Astragalin	*Allium ursinum, Allium sativum, Cassia alata, Cuscuta chinensis, Phytolacca americana*	Flavanoid	448.38	6	13	−0.779	190.508	2
11	Epitheaflavin monogallate	*Camellia sinensis, Chamomilla recutita* and *Mentha pepperita*	Phenolic	716.60	10	13	0.183	300.916	3
12	Procyanidin B2	*Vitis vinifera*	Catechins	578.52	10	10	0.611	223.603	3
13	Quercitrin	*Euphorbia hirta*	Flavanoid	448.38	6	12	−0.57	196.616	2
14	Theaflavin 3,3′-digallate	*Camellia sinensis*	Polyphenol	868.71	13	20	−1.280	376.573	3
15	Procyanidin A1	*Vitis vinifera*	Catechins	576.51	9	10	0.621	208.686	3
16	Curcumin	*Curcuma longa*	Polyphenol	368.38	2	7	2.822	112.459	0
**Limit as per Qikprop module of Schrödinger**				130–725	0–6	2–20	−2 to 6.5	7–200	0-1

a *MW, molecular weight; HBA, hydrogen-bond acceptor atoms; HBD, hydrogen-bond donor atoms; QPlogPo/w, predicted octanol/water partition coefficient; PSA, polar surface area; Green boxes indicate the value that falls under the permissible limit*.

**Table 4:** Predicted pharmacokinetic parameters of phytoconstituents^a^.

**Sr. No**.	**Name**	**Volume**	**QPlogS**	**QPlogHERG**	**QPPCaco**	**QPlogBB**	**%HOA**
1	Procyanidin A3	3,399.108	−5.42	−8.405	0	−12.1	0
2	Acetoside	1,748.392	−2.8	−6.537	0.651	−5.723	0
3	Rutin	1,603.123	−2.605	−5.757	0.368	−5.29	0
4	Solanine	2,316.323	−1.954	−5.488	20.028	−2.318	10.072
5	Procyanidin A4	1,530.546	−4.329	−6.23	0.692	−4.651	0
6	Procyanidin B4	1,529.894	−3.867	−5.507	1.898	−3.749	0
7	Hypericin	1,250.74	−4.713	−5.024	11.926	−2.638	32.557
8	Quercetagetin	882.17	−2.554	−4.894	9.181	−2.736	30.003
9	Procyanidin	1,566.613	−4.176	−6.43	0.476	−4.956	0
10	Astragalin	1,187.782	−2.524	−5.176	11.203	−2.963	15.249
11	Epitheaflavin monogallate	1,787.948	−4.777	−6.144	0.137	−5.67	0
12	Procyanidin B2	1,487.44	−3.509	−5.252	3.819	−3.323	2.066
13	Quercitrin	1,202.959	−2.955	−5.195	7.312	−3.14	13.152
14	Theaflavin 3,3′-digallate	2,132.824	−4.225	−6.519	0.014	−7.218	0
15	Procyanidin A1	1,506.237	−4.608	−6.338	1.974	−4.051	0
16	Curcumin	1,215.586	−4.58	−6.315	155.494	−2.245	82.695
**Limit as per Qikprop module of Schrödinger**		500–2,000	−6.5 to 0.5	Above −5	< 25 poor, > 500 great	−3 to 1.2	< 25 poor, > 80 high

a *QPPCaco, caco-2 cell permeability in nm/s; QPlogBB, brain/blood partition coefficient; QPlogHERG, Predicted IC_50_ value for blockage of HERG K+ channels; QPlogS, predicted aqueous solubility; % HOA, human oral absorption on 0–100% scale; Green boxes indicate the value that falls under the permissible limit*.

Though the compounds showed potential dual enzyme inhibitory actions, most of the compounds did not follow Lipinski's Rule of Five. From the data of Table 3, only quercetagetin and curcumin did not violate the rule. For the rest of the compounds, one can modify the structure in such a way so that it will not reflect their enzyme inhibitory activity but improve the physicochemical properties.

On account of poor ADMET properties, many drug candidates fail in the clinical trials. These late-stage failures drastically contribute to the enhancement of cost for the drugs. Hence, ADMET prediction plays a crucial role in drug discovery and development.

QPCaco-2 is indicative of the oral absorption of a drug. It assesses the apparent gut–blood barrier permeability. Values above 500 predict high oral absorption. None of the compounds listed in Table 4 has higher oral absorption when predicted *in silico*. Curcumin showed moderate oral absorption. Similarly, the percentage of human oral absorption value supports the prediction of oral bioavailability. Curcumin has 82.69% HOA, which is the indication of its high oral bioavailability relatively. QPlogS predicts aqueous solubility, which is again a kind of assessment for oral absorption. All the compounds fall in the range.

QPlogBB indicates the ability to permeate the blood brain barrier (BBB), which is a mandatory parameter for CNS active drugs; for other than CNS active drugs, if these values did not follow in the range, the compound can cause CNS toxicity. Most of the listed compounds will not cross the BBB based on their QPCaco-2 values.

HERG encodes a potassium ion (K+) channel, which is implicated in fatal arrhythmia known as torsade de pointes or the long QT syndrome. This channel contributes to the electrical activity of the heart thereby directing the heart beat responsible for cardiac toxicity of the molecular target. So, QPlogHERG predicts the cardiac toxicity of the compounds. The recommended range for it is above −5. Almost all the phytoconstituents fall in the range except quercetagetin.

### Molecular Dynamic Simulations

In order to understand the time-dependent stability of the complexes between the promising molecules and Mpro/Spike protein, a molecular dynamic (MD) study was carried out. The MD study was performed for a period of 10 ns using the Gromacs2020.1 package. Here, the docked pose of the ligand–receptor was considered as the reference frame for the MD study, and various statistical parameters such as RMSD-P, RMSF-P, RMSD-L (*P* = protein, *L* = ligand), and H-bonding were determined (Supplementary Figures 1–3).

The protein RMSD-P is analyzed to understand the degree of movement of the protein or atoms while putting the ligand in the active site and proposing the structural stability, deviation, and conformations of the protein over the simulation time. The RMSD-P for Mpro in complexation with curcumin (42) was in the range of 0.08–0.3 with an average of 0.19 nm (Supplementary Figure 1A). This suggests the stability of the protein while having curcumin (42) in the active site of Mpro over this simulation time. Despite having high flexibility, curcumin (42) exhibited RMSD-L values in the range of 0.18–0.65 nm consistently except for the time duration from 4.72 to 6.05 where a sharp shoot in RMSD-L was observed up to 1.1 nm, and it followed normalcy for the remaining period of simulation (Supplementary Figure 1B). RMSF explains the residual mobility and integrity of the structure. The observed RMSF-P for the residues up to 300 was below 0.3 nm, whereas the protein tail above residue number 300 showed fluctuation up to 0.56 nm (Supplementary Figure 1C). Hydrogen bonds between curcumin (42) and Mpro protein over the period of analysis were determined with Gromacs g_hbond utility. A maximum of five hydrogen bonds were observed during MD simulation, whereas two to three hydrogen bonds were observed consistently throughout the simulation time (Supplementary Figure 1D). The short-range electrostatic (Coul-SR) and van der Waals/hydrophobic (LJ-SR) interaction energies between protein and compound (42) explained promising electrostatic as well as hydrophobic interactions. The average values of Coul-SR −44.55 ± 5.0 kJ/mol and LJ-SR −114.97 ± 6.1 kJ/mol were observed. This suggests that the role of hydrophobic interaction was more important than the electrostatic interactions in stabilizing the complex.

A similar evaluation was done for Mpro with solanine (4). Here, the RMSD-P value was observed in the range of 0.1–0.28 nm with an average of 0.19 nm (Supplementary Figure 2A). Despite having multiple rotatable bonds, the RMSD-L was observed in the range of 0.13–0.37 with an average value of 0.25 nm (Supplementary Figure 2B). The observed RMSF-P for the residues up to 300 was below 0.25 nm, whereas the protein tail above residue number 300 showed fluctuation up to 1.0 nm (Supplementary Figure 2C). Overall, this ligand–receptor complex showed a maximum of eight hydrogen bonds, whereas five H-bonds were consistently observed over the period of time (Supplementary Figure 2D). The short-range electrostatic (Coul-SR, energy: −264.7 ± 5.1 kJ/mol) and van der Waals/hydrophobic (LJ-SR, energy: −173.9 ± 3.6 kJ/mol) interaction energies suggested promising interactions between the ligand and the protein. The contribution of electrostatic interactions was found to be higher than that of the hydrophobic interactions.

For solanine (4) in complexation with spike protein, the RMSD-P values in the range of 0.08–0.17 nm (average 0.12 nm) explained the stability of the protein while having solanine (4) in the active site (Supplementary Figure 3A). Solanine (4) exhibited RMSD-L values in the range of 0.11–0.42 nm consistently except for the time duration from 9.06 to 9.76 where a sharp shoot in RMSD-L was observed up to 0.68 nm, and it followed normalcy for the remaining period of the simulation (Supplementary Figure 3B). Here, the overall RMSF-P value below 0.35 nm also supports the stability of the protein in the presence of the ligand in the active site (Supplementary Figure 3C). This ligand–receptor complex showed a maximum of six hydrogen bonds, whereas three to four H-bonds were consistently observed over the period of time (Supplementary Figure 3D). The short-range electrostatic (Coul-SR, energy: −91.30 ± 9.5 kJ/mol) and van der Waals/hydrophobic (LJ-SR, energy: −97.88 ± 2.3 kJ/mol) interaction energies suggested promising interactions between the ligand and the protein.

### Discussion

SARS-CoV-2 Mpro and Spike Glycoprotein RBD bound to ACE2 inhibitory activities were evaluated for a diverse class of compounds that include: terpenoids, coumarins, flavonoids, glycosides, phenols and polyphenols, catechins, etc. Of all the groups of flavonoids studied, flavonol glycosides like rutin, astragalin, quercitrin, baicalin, myricetin-3-glucoside, amentoflavone, etc., showed better docking scores than the other groups of this category. Flavones (apigenin, chrysin, and luteolin) and soyabean isoflavones (daidzein and genistein) showed moderate binding affinity for MPro. Further, of the flavan 3-ols (catechins/procyanidins), complex oligomeric procyanidins (procyanidin A3, procyanidin A4, procyanidin A1, and procyanidin B3) were found to be having very good binding scores. Simple phenolic glycosides like salicin and arbutin have scores of −8.448 and −7.137, respectively. Among the furanoid diterpene glycosides found in *Tinospora cordifolia*, cordifoliside D showed a good score against Mpro. Anthraquinones, hypericin, and emodin 8-glucoside are good candidates for targeting MPro. Solanine, a steroidal glycoside, rutin, and acetoside are among the best compounds as far its binding ability with Mpro and spike RBD are concerned. Curcumin and acetoside covalently inhibit Mpro. Curcumin also passes Lipinski's rule of five and possesses good pharmacokinetic parameters when investigated *in silico*. The MD simulation study showed the time-dependent stability of ligand–receptor complexes (curcumin with Mpro, and solanine with Mpro and spike RBD) for period of 10 ns.

## Conclusion

Among the total of 170 phytoconstituents screened virtually, 59 phytochemicals showed comparatively good Mpro inhibition. Two of them (curcumin and acetoside) showed covalent bonding with Cys145 of Mpro. The same set of phytoconstituents were docked against Spike glycoprotein RBD bound to ACE2. About 10 bioactive compounds were found to inhibit spike glycoprotein RBD as well. While investigating the overall docking result, it was found that solanine, rutin, and acetoside potentially inhibit both the receptors so they can serve as dual receptor inhibitors. However, these compounds did not possess comparatively good ADMET parameters. One can modify their structures to develop good ADMET parameters. From the above study, Curcumin was found to possess good Mpro inhibition as well as better ADMET properties. Solanine was further assessed for their solanine–Mpro receptor complex and solanine-spike RBD complex stability with MD simulation study, which makes it a dual receptor inhibitor.

## Data Availability Statement

The raw data supporting the conclusions of this article will be made available by the authors, without undue reservation.

## Author Contributions

MS and MC conceptualized the research work and reviewed and edited the manuscript. DT collected and analyzed the data and wrote the manuscript. All authors contributed to the article and approved the submitted version.

## Conflict of Interest

The authors declare that the research was conducted in the absence of any commercial or financial relationships that could be construed as a potential conflict of interest.
