# Supplementary material for: In silico Screening of Natural Compounds as Potential Inhibitors of SARS-CoV-2 Main Protease and Spike RBD: Targets for COVID-19
Source: Front Mol Biosci. 2021 Jan 19;7:599079. doi: 10.3389/fmolb.2020.599079 (PMC7852456; doi:10.3389/fmolb.2020.599079)
Supplement: Supplementary file 1 [file Data_Sheet_1.PDF]

In silico Screening of Natural Compounds as Potential Inhibitors of SARS-CoV-2 Main Protease and Spike RBD, targets for COVID-19

Divya M. Teli[1], Mamta B. Shah[2], Mahesh T. Chhabria[1*]

[1]Department of Pharmaceutical Chemistry, L. M. College of Pharmacy, Navarangpura, Ahmedabad, Gujarat-380009, India.

[2]Department of Pharmacognosy, L. M. College of Pharmacy, Navarangpura, Ahmedabad, Gujarat-380009, India.

*Corresponding author:
Mahesh T. Chhabria
Department of Pharmaceutical Chemistry,
L. M. College of Pharmacy,
Navarangpura, Ahmedabad, Gujarat-380009, India.
Email address: mahesh.chhabria@lmcp.ac.in

Supporting information

Molecular dynamic (MD) simulation study

Following are the figures for MD simulation of ligand-receptor complexes (curcumin with Mpro, solanine with Mpro and spike RBD) over a period of 10 ns.

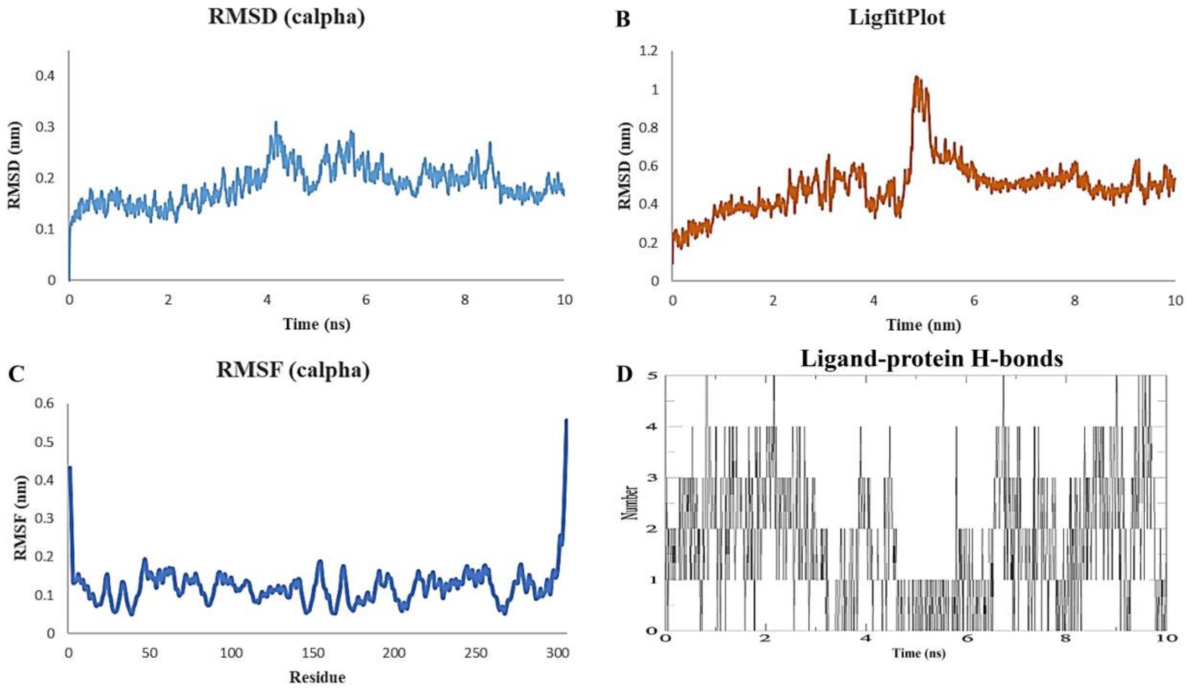

FIGURE S1 (A) RMSD-P, (B) RMSD-L, (C) RMSF-P and (D) H-bond plots for Mpro with curcumin (**42**).

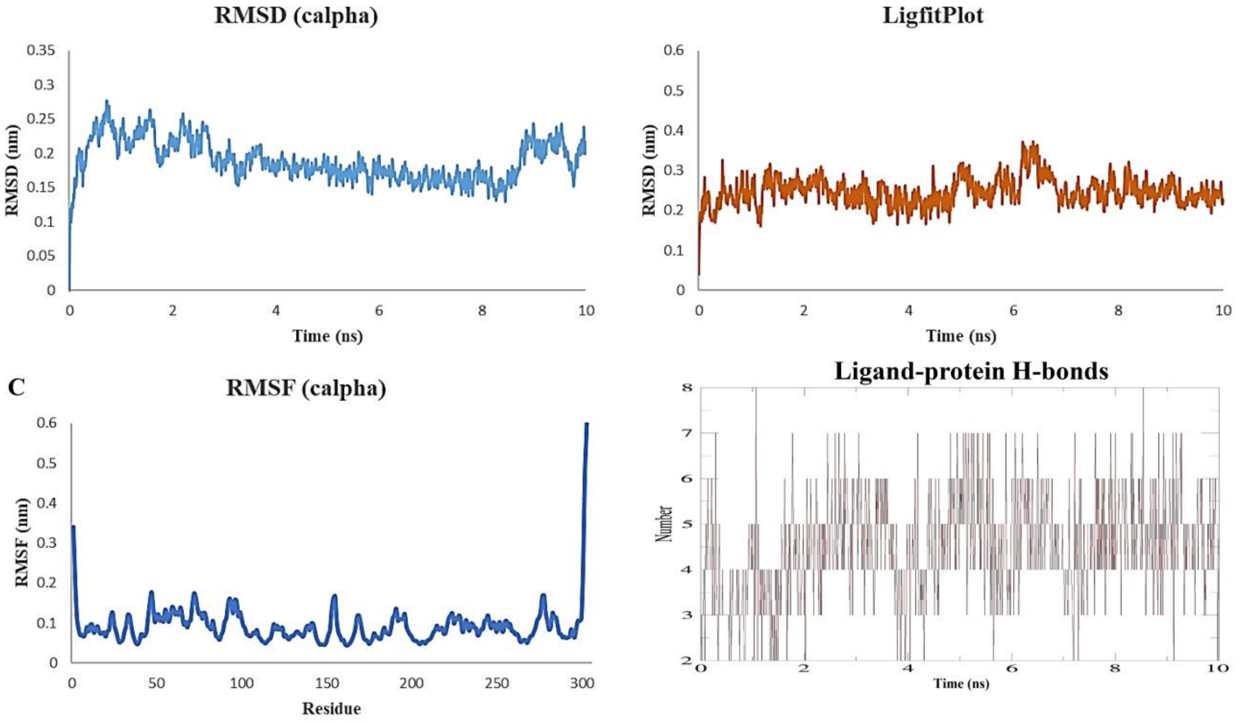

FIGURE S2 (A) RMSD-P, (B) RMSD-L, (C) RMSF-P and (D) H-bond plots for Mpro with solanine (**4**).

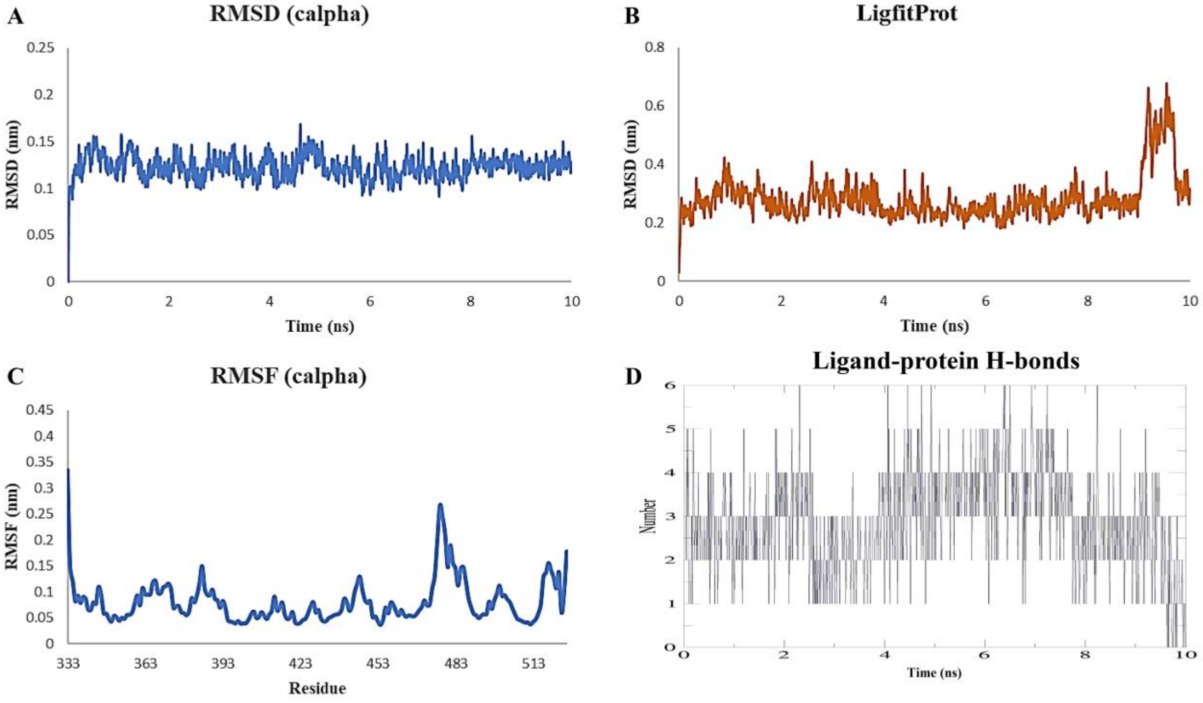

FIGURE S3 (A) RMSD-P, (B) RMSD-L, (C) RMSF-P and (D) H-bond plots for Spike protein with solanine (**4**).
